# d-amino acids signal a stress-dependent run-away response in *Vibrio cholerae*

**DOI:** 10.1038/s41564-023-01419-6

**Published:** 2023-06-26

**Authors:** Oihane Irazoki, Josy ter Beek, Laura Alvarez, André Mateus, Remy Colin, Athanasios Typas, Mikhail M. Savitski, Victor Sourjik, Ronnie P.-A. Berntsson, Felipe Cava

**Affiliations:** 1 The Laboratory for Molecular Infection Medicine Sweden (MIMS), Umeå Center for Microbial Research (UCMR), Science for Life Laboratory (SciLifeLab), Department of Molecular Biology, Umeå University, Umeå, Sweden; 2 Department of Medical Biochemistry and Biophysics, Umeå University, Umeå, Sweden; 3 Wallenberg Centre for Molecular Medicine, Umeå University, Umeå, Sweden; 4 Genome Biology Unit, European Molecular Biology Laboratory, Heidelberg, Germany; 5 Max Planck Institute for Terrestrial Microbiology, and Center for Synthetic Microbiology (SYNMIKRO), Marburg, Germany

**Keywords:** Bacterial physiology, Bacteriology

## Abstract

To explore favourable niches while avoiding threats, many bacteria use a chemotaxis navigation system. Despite decades of studies on chemotaxis, most signals and sensory proteins are still unknown. Many bacterial species release d-amino acids to the environment; however, their function remains largely unrecognized. Here we reveal that d-arginine and d-lysine are chemotactic repellent signals for the cholera pathogen *Vibrio cholerae*. These d-amino acids are sensed by a single chemoreceptor MCP_DRK_ co-transcribed with the racemase enzyme that synthesizes them under the control of the stress-response sigma factor RpoS. Structural characterization of this chemoreceptor bound to either d-arginine or d-lysine allowed us to pinpoint the residues defining its specificity. Interestingly, the specificity for these d-amino acids appears to be restricted to those MCP_DRK_ orthologues transcriptionally linked to the racemase. Our results suggest that d-amino acids can shape the biodiversity and structure of complex microbial communities under adverse conditions.

## Abstract (web summary)

d-arginine and d-lysine are chemorepellent molecules sensed by a novel chemotaxis receptor in *Vibrio cholerae* that trigger a run-away response under adverse conditions.

## Main

Most bacteria can sense and respond to a wide variety of signals to thrive in changing environments. One such adaptive response is chemotaxis, by which motile bacteria monitor changes in local concentrations of certain substances and initiate a signalling cascade that, over time, biases bacterial movement towards favourable conditions (for example, nutrients) or away from toxic compounds^1^.

Albeit species-specific components exist, the core of the chemotaxis signalling pathway consists of chemoreceptors, known as methyl-accepting chemotaxis proteins (MCPs), the adaptor protein CheW, the histidine kinase CheA and the response regulator CheY. In some species such as *Escherichia coli*, the MCPs form a stable ternary sensory complex with CheW and CheA that typically clusters at cell poles. Upon ligand binding or other environmental perturbations, the MCPs change conformation and modulate CheA kinase activity with the help of CheW. CheA in turn phosphorylates CheY, thereby allowing it to bind to the flagellar motor to induce clockwise rotation and, thus, cell tumbling^2,3^. To ensure a rapid response to chemotactic stimuli, the phosphorylation of CheY can be controlled by a specific phosphatase^4^. Although the chemotaxis signalling core components are highly conserved among bacteria and archaea, the number and type of chemoreceptors are highly variable between species and their specificities remain largely uncharacterized^5–7^.

Compared to *E. coli*, which has a single chemosensory system and 5 MCPs, the facultative pathogen *Vibrio cholerae* has a very sophisticated chemotaxis system organized into three sets of chemosensory pathways (Che systems I/F9, II/F6 and III/F7) and at least 45 chemoreceptors^8^. Thus far, only system II/F6 has been demonstrated to control motility^9^, while the function of the others remains unclear. The high number of MCPs has been suggested to reflect the complexity of *V. cholerae’s* life cycle^10^; however, except for a handful of input signals (for example, l-amino acids^11^, taurine^12^, oxygen^13^) there is almost no information about the ligand specificity of most MCPs.

**Fig. 1:**
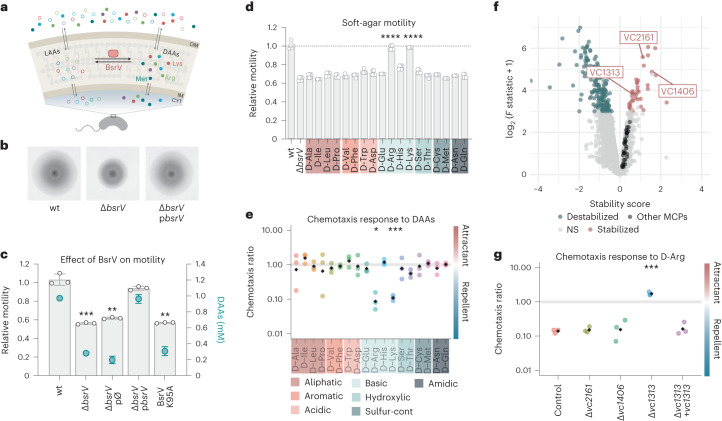
d-Arg and d-Lys signal a chemorepellent response in *V. cholerae* through the putative MCP VC1313. **a**, Schematic representation of DAA racemization. The most abundant amino acids produced are shown (open circles, l-amino acids; filled circles, d-amino acids). OM, outer membrane; IM, inner membrane; CYT, cytoplasm. **b**, Representative images of *V. cholerae* wild type (wt) and Δ*bsrV* mutant expanding in 0.3% soft agar. Images are representative of experiments repeated at least three times. **c**, Motility and d-amino acid production analysis of BsrV derivatives; grey bars indicate relative motility compared to wt or Δ*bsrV* knockout mutant, catalytic site mutant (BsrV K95A) and the complemented strain harbouring *bsrV* under an aTc inducible promoter (p*bsrV*); green dots represent the amount of secreted d-amino acids. pØ, empty plasmid. Error bars represent mean ± s.d. of 3 biologically independent replicates. Significant differences (paired *t*-test) are indicated by **P < 0.01 or ***P < 0.001. **d**, Motility of Δ*bsrV* mutant in soft-agar plates that are chemically complemented with 5 mM d-amino acids are shown relative to the wild-type strain. Error bars represent mean ± s.d. of 6 biologically independent replicates examined over 2 independent experiments. Significant differences (one-way analysis of variance (ANOVA)) are indicated by ****P < 0.0001. **e**, Chemotactic response to 0.1 mM d-amino acids in capillary assays. Chemotaxis ratio (CR) was calculated relative to the control capillary containing no stimulus. CR > 1, attractants; CR < 1, repellents; CR = 1, no response. Black diamonds represent the mean of 3 independent biological replicates. Significant differences (unpaired *t*-test) are indicated by *P < 0.05 or ***P < 0.001. **f**, Thermal proteome profiling analysis of *V. cholerae* cell extracts exposed to d-Arg. Stabilized and destabilized proteins in the presence of d-Arg are highlighted in red and green, respectively. From the 45 MCPs found in *V. cholerae’s* genome (black), only 3 interacted with d-Arg: VC2161, VC1313 and VC1406. **g**, Chemotactic response of selected MCP candidates to d-Arg. Double mutants (Δ*bsrV Δmcp*) were constructed and the ability to respond to d-Arg was tested by capillary assays. Δ*bsrV* strain was used as background. LAA, l-amino acids; DAA, d-amino acids. Black diamonds represent the mean of 3 independent biological replicates. Significant differences (unpaired *t*-test) are indicated by ***P < 0.001. Source data

*V. cholerae* releases millimolar concentrations of diverse d-amino acid (DAAs) into the environment^14,15^ (Fig. 1a). DAAs are produced from their l-amino acid (LAA) counterparts by the broad-spectrum racemase BsrV, which is conserved in many bacterial species^14^. These molecules regulate diverse cellular processes (for example, cell wall biogenesis^16,17^, biofilm integrity^18–21^, spore germination^22^ or bacteria–bacteria interactions^15^) but overall, the physiological role of extracellular DAAs is mainly dependent on each specific d-amino acid type and bacterial species. In *V. cholerae*, it is known that d-Met and d-Leu modulate cell wall biogenesis, but the function of other DAAs is unclear.

## Results

### d-arginine and d-lysine are chemorepellent signals

**Extended Data Fig. 1:**
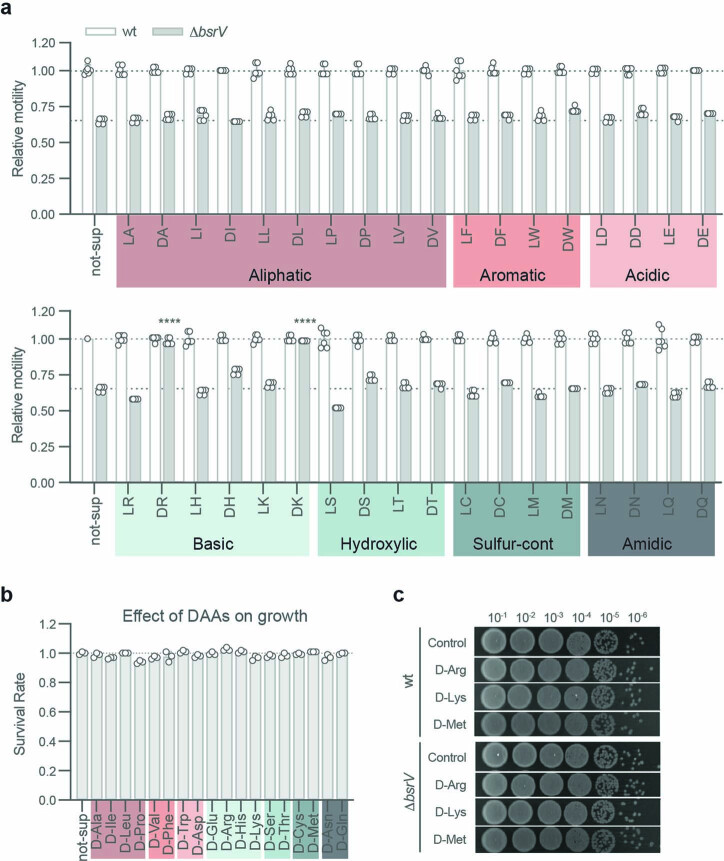
Effect of D-amino acids in motility. **a**, Motility analysis of *V. cholerae* in soft agar in presence of L- and D-amino acids. Grey bars indicate relative motility of BsrV knockout mutant compared to wild-type (wt) both in presence and absence of 5 mM amino acids. **b**, Effect of D-amino acids (DAAs) in growth. Survival rate was calculated as relative maximum growth in late stationary phase using the not supplemented culture as reference. Cultures were supplemented with 5 mM DAAs. **c**, Spot assay for the indicated *V. cholerae* strains. Cells were grown in TB until stationary phase, serially diluted and spotted on LB agar plates supplemented with 5 mM DAAs. Error bars in **a** and **b** represent mean values ± SD of 6 and 3 biologically independent replicates. Significant differences (One-way ANOVA, p-values were adjusted for multiple comparison using Bonferroni-Dunn method) are indicated by **** (p < 0.0001). Source data

While screening for DAA-specific phenotypes, we found that a *V. cholerae* strain defective in d-amino acid production (Δ*bsrV*) exhibited a 40% motility reduction compared to wild-type cells in soft-agar plates. This reduction was fully restored upon ectopic genetic complementation with the *bsrV* racemase (Fig. 1b,c). Δ*bsrV’s* motility defect was recapitulated by a catalytically inactive mutant (BsrV K95A, ref. ^23^), thus suggesting that this phenotype was directly associated with BsrV’s activity to produce d-amino acids. To ascertain that the absence of secreted d-amino acids accounted for the motility defect that we found, we chemically complemented the Δ*bsrV* mutant by supplementing the soft-agar plates with innocuous concentrations of individual DAAs. Their corresponding l-enantiomers were used as controls (Extended Data Fig. 1). We found that only d-arginine (d-Arg) and d-lysine (d-Lys) restored the motility of the Δ*bsrV* mutant to wild-type levels (Fig. 1d), suggesting that these specific DAAs play a specific role in *V. cholerae’s* ability to spread in soft agar.

**Extended Data Fig. 2:**
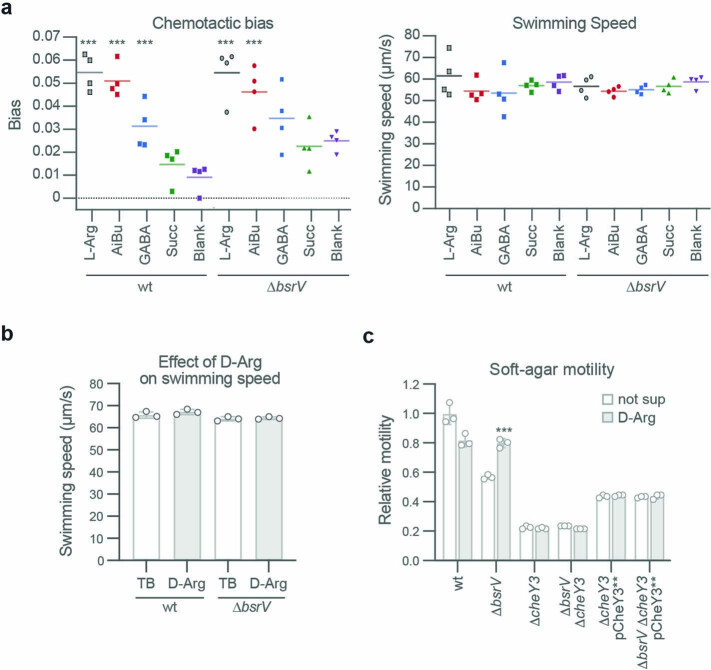
Comparison of chemotactic ability of *V. cholerae* wild-type and Δ*bsrV* strains. **a**, Chemotactic ability of Δ*bsrV* mutant. Both chemotactic bias and swimming speed were analysed in presence of several known chemoeffectors to compare the behaviour of wild-type (wt) and Δ*bsrV* mutant cells. Chemotaxis was measured in microfluidic devices, monitoring cell motion from a reservoir filled with cells in motility buffer to a reservoir filled only with 100 µM of indicated compound in motility buffer. Blank, no attractant in second reservoir; L-Arg, L-arginine; AiBu, α-aminoisobutiric acid; GABA, γ-aminobutyric acid; Succ, succinate. Each data point represents the average over the 3 time points for a given experiment (n = 4) and the line represents the mean. Significant differences (unpaired *t*-test) are indicated by *** (p < 0.001) and was evaluated relative to the blank for the given strains. **b**, Swimming speed analysis of *V. cholerae* grown in presence of D-Arg. Cells were grown in TB (± 500 μM D-Arg) and transferred to fresh TB for swimming speed measurements. Each point represents the mean and error bars indicate the SD over 3 biological replicates. **c**, Motility analysis of non-chemotactic *V. cholerae* cells in presence and absence of D-Arg. Relative motility on soft-agar plates supplemented or not with 5 mM D-Arg of several strains compared to wild-type (wt). Δ*bsrV* and Δ*cheY3* strains are included as controls. Error bars in **c** represent mean values ± SD of 3 biologically independent replicates. Significant differences (unpaired *t*-test, adjusted for multiple comparison using Bonferroni-Dunn method) are indicated by *** (p < 0.001). See source data. Source data

To narrow down the role of d-Arg and d-Lys in *V. cholerae’s* motility, we first assessed the integrity of the flagellum. However, Δ*bsrV* cells showed no significant differences in flagellum composition or structure compared to the wild type (Supplementary Fig. 1). As motility is functionally related to chemotaxis, we reasoned that d-Arg and d-Lys modulate *V. cholerae’s* chemotactic response. In this line, we tested the activity of diverse DAAs in capillary assays. We used the Δ*bsrV* genetic background (control hereafter) to prevent BsrV-mediated racemization of the amino acids during the assays. Importantly, this strain has wild-type chemotactic and swimming ability towards previously characterized stimuli (for example, l-Arginine, α-aminoisobutyric acid; γ-aminobutyric acid, succinate) (Extended Data Fig. 2). Interestingly, we found that both d-Arg and d-Lys promote repulsive chemotactic responses in *V. cholerae* Δ*bsrV*, while the other DAAs did not affect the bacteria (Fig. 1e). To confirm that the motility defect of *V. cholerae* Δ*bsrV* is exclusively related to chemotaxis signalling and not due to a change in *V. cholerae’s* swimming capacity, we used a permanently active phosphomimetic *cheY3*^D16K,Y109K^ mutant (hereafter referred to as CheY3**). As previously described^24,25^, CheY3** cells are non-chemotactic because they are locked in tumbling mode but maintain the ability to swim. Our results show that the spread of a Δ*cheY3* strain complemented with CheY3** on soft-agar plates was unaffected by d-Arg (Extended Data Fig. 2c), thus indicating that this DAA indeed does not regulate *V. cholerae* swimming. Collectively, these results show that d-Arg and d-Lys sensing induces a repellent chemotactic response in *V. cholerae*.

### VC1313 is the chemoreceptor of d-Arg and d-Lys

**Extended Data Fig. 3:**
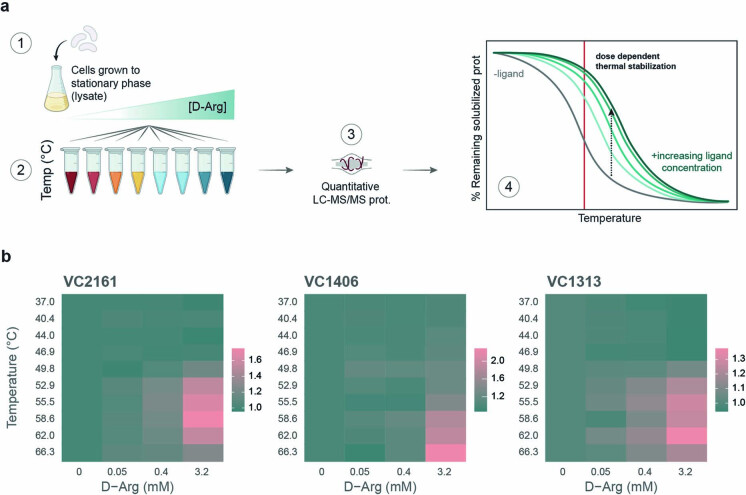
Thermal proteome profiling. **a**, Schematic representation of Thermal Proteome Profiling (2D-TPP) experimental setting. 4 different conditions were tested: 0, 0.05, 0.4, 3.2 mM D-Arg. Cell extracts were prepared and treated with a range of D-Arg concentrations (1); the samples were heated to a range of temperatures (2); then soluble proteins fraction was extracted and digested to analyze by mass-spectrometry (3); finally the effect of ligand on thermal stability of each protein was evaluated by comparing the remaining soluble fraction at each ligand concentration with the untreated control for each temperature (4). **b**, Heatmaps for selected candidates targeting D-Arg in *V. cholerae*. The legend represents the log_2_-transformed fold-change protein abundance for a given temperature and D-Arg concentration relative to the no D-Arg control.

**Extended Data Fig. 4:**
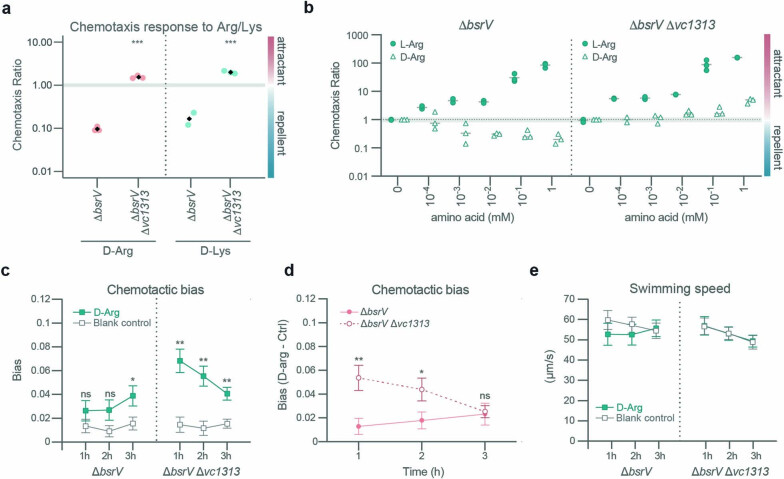
Chemotactic ability of *V. cholerae vc1313* mutant towards Arg. **a**, Comparison between D-Arg and D-Lys chemotaxis responses in *V. cholerae*. **b**, Chemotaxis response to L- and D-Arg increasing concentrations for *V. cholerae* Δ*bsrV* and Δ*bsrV* Δ*vc1313* double mutant. Significant differences (unpaired t-test) are indicated by *** (p < 0.001), n = 3. **c**, Chemotactic bias of *V. cholerae* Δ*bsrV* and Δ*bsrV* Δ*vc1313*, measured in microfluidics devices, towards a reservoir containing either 500 µM D-Arg in motility medium or only motility medium (blank control), 1, 2 and 3 h after the preparation of the device. **d**, Difference between the chemotactic bias (that is, the ratio *v*_*ch*_/*v*_*0*_ of the chemotactic drift velocity *v*_*ch*_ over the cell swimming speed *v*_*0*_, see Methods) towards D-Arg and the corresponding control of the experiments of (b) as a function of the time since the preparation of the microfluidic device. **e**, Population averaged swimming speed measured in the experiments of (b, c). Each data point of in **c, d** and **e** represents the mean and error bars ± SEM of n = 6 biologically independent replicates. Significant differences (unpaired t-test) are indicated by *ns* (not-significant), * (p < 0.05) ** (p < 0.01) or *** (p < 0.001). See source data. Source data

The *V. cholerae* chemotaxis machinery comprises at least 45 putative MCPs^8^, some of which are known to be functionally redundant (for example, Mlp24 and Mlp37, which sense several LAAs)^11,12^. Therefore, to screen for MCP candidates sensing d-Arg and d-Lys, we opted for two-dimensional thermal proteome profiling (2D-TPP) (refs. ^26,27^) over mutagenesis. TPP is based on the principle that protein thermal stability can be affected by interactions with ligands^28^. In our TPP experiment, we treated *V. cholerae* cells with different concentrations of d-amino acids and quantified the resulting changes in protein thermal stability using mass spectrometry (Extended Data Fig. 3a). Considering the structural similarity and comparable chemotactic responses of d-Arg and d-Lys, we focused on d-Arg for our study. Our results revealed ~223 proteins affected in their thermal stability (false discovery rate, FDR <0.01) by d-Arg (Fig. 1f, Extended Data Fig. 3b, and Supplementary Tables 1 and 2). Some 185 proteins presented a decreased thermal stability, while 38 displayed increased thermal stability, including three putative MCPs: VC2161, VC1406 and VC1313. To further investigate whether any of these chemoreceptors reacts to d-Arg, we generated individual MCP knockouts in the Δ*bsrV* background and examined their chemotactic response to d-Arg. Remarkably, we found that the d-Arg response of the Δ*vc2161* and Δ*vc1406* strains was comparable to that of the control, while the Δ*vc1313* mutant did not respond to d-Arg. Notably, the d-Arg response in this mutant was fully restored upon genetic complementation with *vc1313* (Fig. 1g). We found that Δ*bsrV* Δ*vc1313* cells were also unresponsive to d-Lys (Extended Data Fig. 4a), indicating that this MCP senses both DAAs. Additional capillary and microfluidic-based assays^29^ with a broader range of amino acid concentrations narrowed VC1313’s repellent response to d-Arg concentrations in the 0.01 mM to 1 mM range (Extended Data Fig. 4). Interestingly, 1 mM d-Arg showed a slightly positive chemotactic response in Δ*bsrV* Δ*vc1313* cells. This suggests that alternative lower-affinity chemoreceptors sense d-Arg as an attractant. However, our results show that *vc1313* encodes the MCP that senses d-Arg and d-Lys to initiate a repulsive chemotactic response in *V. cholerae*; therefore, we renamed this protein as MCP_DRK_ for MCP sensing d-Arg (R) and d-Lys (K).

### RpoS sigma factor regulates MCP_DRK_

**Extended Data Fig. 5:**
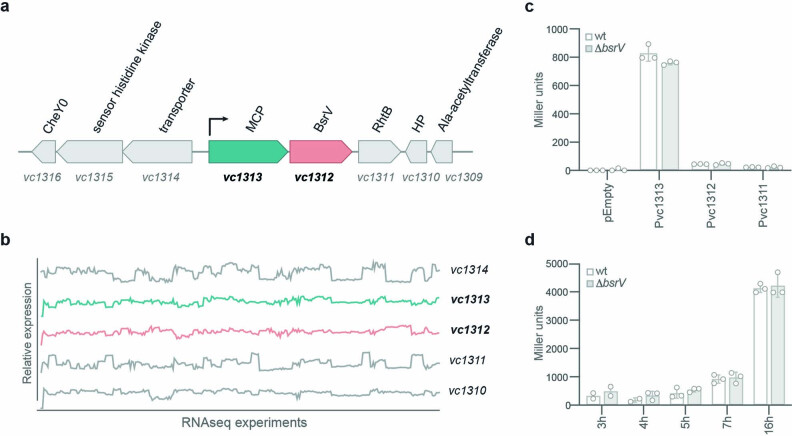
MCP_DRK_ genetic context and promoter transcriptional activity. **a**, Genetic context of *vc1313*. **b**, Relative expression of *vc1313-vc1312* and adjacent genes across 307 available *V. cholerae* RNAseq experiments. **c**, Transcriptional activity of the putative promoter regions of the genes forming the operon (*vc1313, vc1312* and *vc1311*) in wild-type (wt) and Δ*bsrV* mutant backgrounds. β-galactosidase assay was performed in cultures carrying pCB192N-derivatives grown for 8 hours at 37 °C in LB. β-galactosidase activity is measured in Miller units. **d**, Transcriptional activity of the Pvc1313 putative promoter region at different timepoints. β-galactosidase assay was performed in cultures of wild-type (wt) and Δ*bsrV* mutant backgrounds carrying pCB192N-Pvc1313, grown at 37 °C in LB for the indicated time. β-galactosidase activity is measured in Miller units. Error bars in **c** and **d** represent mean values ± SD of 3 biologically independent samples.

The *mcp*_*DRK*_ locus is localized in the vicinity of *vc1312*, which encodes BsrV—the broad-spectrum racemase that produces d-Arg and d-Lys (Extended Data Fig. 5a). Analysis of the expression profiles across different publicly available RNAseq datasets for *V. cholerae* strongly suggests that these two genes are co-regulated and could form an operon (Extended Data Fig. 5b). Using promoter-probe plasmids, we found a promoter region upstream of *vc1313* (Pvc1313) but not of *vc1312* (Extended Data Fig. 5c). Moreover, Pvc1313 activity increases in stationary phase, consistent with a previous study showing that BsrV is induced under the same conditions, in an RpoS-dependent fashion^30^ (Extended Data Fig. 5d).

**Fig. 2:**
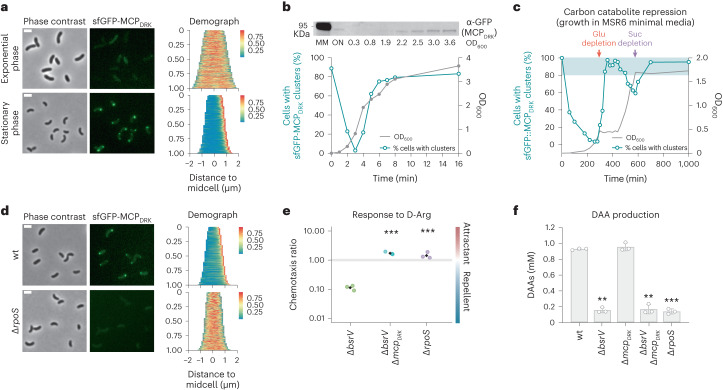
d-Arg production and chemotactic response are coordinated by RpoS. **a**, Left: localization of sfGFP-MCP_DRK_ expressed from its native locus and promoter at exponential and stationary phase. Representative micrographs of 3 independent replicates are shown; at least 3 images were acquired per timepoint. Scale bar, 2 μm. Right: demographic analysis of the fluorescence intensity of sfGFP-MCP_DRK_ relative to cell length (*n* > 500 cells). The colour bar represents fluorescence intensity. **b**, Bottom: growth-phase dependent subcellular localization of sfGFP-MCP_DRK_. Filled grey circles, OD_600 _ of *V. cholerae* grown in TB; open tradewind-blue circles, percentage of cells with sfGFP-MCP_DRK_ polar foci. Top: western blot against sfGFP-MCP_DRK_ using anti-GFP-specific antibody at the indicated OD_600_. Samples were normalized and loaded with equal protein amount. **c**, Growth curve of *V. cholerae* grown in MSR6 minimal medium supplemented with 0.04% w/v d-glucose and 0.4% w/v succinate (grey line) at the indicated timepoints. The corresponding percentage of cells with sfGFP-MCP_DRK_ foci (open tradewind-blue circles) is also indicated. Red (glucose depletion) and purple (succinate depletion) arrows indicate carbon starvation. Quantification of % of cells with polar foci in **b** and **c** was based on a single experiment where >500 cells were counted from 3 different images per timepoint. **d**, Left: micrographs showing the expression of sfGFP-MCP_DRK_ in a Δ*rpoS* mutant background. Representative micrographs of 3 independent replicates are shown; at least 3 images were acquired per timepoint: scale bar, 2 μm. Right: demographic analysis of the fluorescence intensity of sfGFP-MCP_DRK_ relative to cell length. The colour bar represents fluorescence intensity. **e**, Chemotaxis response of Δ*rpoS* mutant to d-Arg. Black diamonds represent the mean of 3 independent biological replicates. Error bars represent mean ± s.d. of 3 biologically independent replicates. Significant differences (unpaired *t*-test) are indicated by ***P < 0.001. **f**, DAA production by the different strains. Significant differences (unpaired *t*-test) are indicated by **P < 0.01 or ***P < 0.001. Source data

To confirm this result, we decided to monitor the expression and localization of MCP_DRK_ labelled with super-folding green fluorescence protein (sfGFP-MCP_DRK_) throughout *V. cholerae* growth. As predicted, cultures inoculated with stationary phase cells displayed polar MCP_DRK_ localization in nearly 90% of the cells. This percentage rapidly dropped as the cells started to grow until no foci were detected at the onset of exponential growth (Fig. 2a). As cultures plateaued, polar foci reappeared, reaching the starting levels. We found a direct correlation between sfGFP-MCP_DRK_ foci and protein levels at different optical densities by western blot (Fig. 2b). This demonstrates that expression of MCP_DRK_ in *V. cholerae* is indeed linked to the stationary phase. As RpoS is highly induced during glucose starvation, we cultured *V. cholerae* on a mixture with glucose and succinate and monitored expression and localization of MCP_DRK_. Similar to other bacteria, *V. cholerae* cells follow a diauxic growth caused by catabolite repression; cells consume d-glucose first, followed by a short pause, then resuming growth by metabolizing succinate until it is spent^30^ (Fig. 2c). We again observed that the stationary phase cells from the inoculum exhibited polar sfGFP-MCP_DRK_ foci in basically all cells, but these vanished when growth started. Interestingly, foci rapidly increased to maximum levels when cells entered the first growth arrest caused by glucose depletion. When cells resumed growth on succinate, foci decreased to ~60% and rose up again when succinate was consumed. These results indicate that the induction and polar localization of MCP_DRK_ respond to growth arrest rather than high population densities.

To confirm that MCP_DRK_’s expression depended on RpoS, we cloned the sfGFP-MCP_DRK_ reporter into a Δ*rpoS* mutant background. In contrast to wild-type stationary phase cultures where most cells displayed foci, the Δ*rpoS* cells showed no polar localization of the MCP (Fig. 2d). Consistently, Δ*rpoS* phenocopied the lack of DAA production and taxis response to d-Arg of the Δ*bsrV* and Δ*mcp*_*DRK*_ mutants (Fig. 2e,f), respectively. These results demonstrate that *V. cholerae* displays a synchronic RpoS-dependent DAA production and MCP_DRK_-chemotactic response during nutrient deprivation stresses that lead to growth arrest.

### MCP_DRK_ forms part of the chemotaxis system II/F6

**Extended Data Fig. 6:**
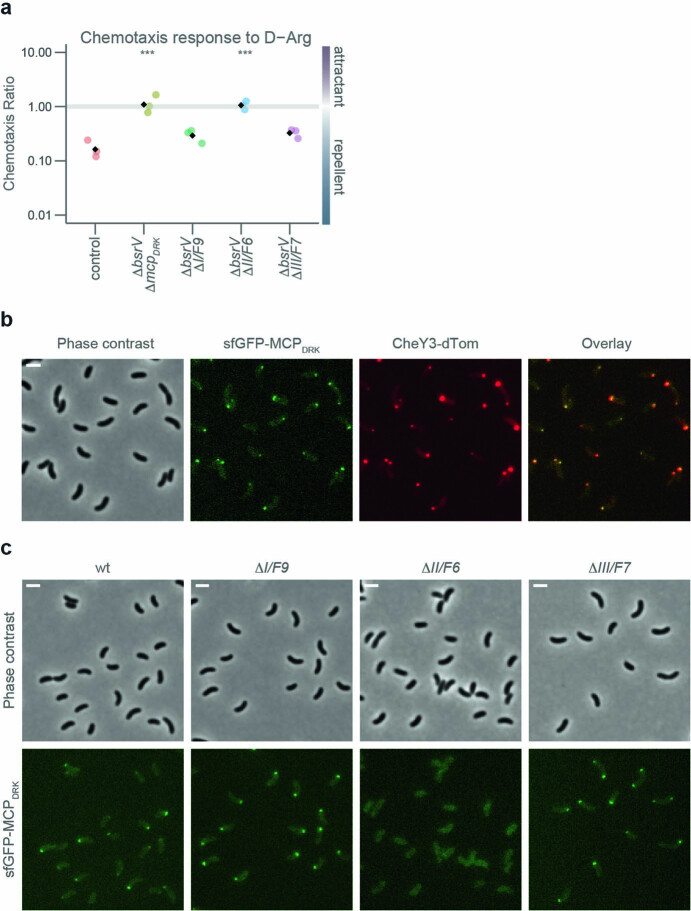
MCP_DRK_ forms part of the chemotaxis F6 system. **a**, Chemotaxis response to D-Arg of chemotaxis system mutants. Δ*I/F9* and Δ*III/F7* showed similar response to D-Arg as control strain. No response was observed for Δ*II/F6* system mutant. Δ*bsrV* strain was used as background. Black diamond represents the mean of 3 independent biological replicates. Significant differences (unpaired t-test) are indicated by *** (p < 0.001), n = 3. **b**, Co-localization of sfGFP-MCP_DRK_ and CheY3-dTtomato (chemotaxis system II/F6) fluorescence reporters during stationary phase. **c**, Localization of sfGFP-MCP_DRK_ in the three chemotaxis systems knockout backgrounds. Representative micrographs of all experiments are shown. Representative micrographs of 3 independent replicates are shown in **b** and **c**, at least 3 images were acquired per condition: scale bar, 2 μm. Source data

Similar to *V. cholerae* chemosensory cluster III/F7 proteins^30^, MCP_DRK_ also forms polar foci during the stationary phase and its expression is RpoS-dependent. However, based on domain length and sequence conservation, MCP_DRK_ belongs to the 40H (heptad) class of chemoreceptors^31^. These are membrane-bound, polarly localized MCPs predicted to signal through the chemotaxis system II/F6—the only one so far related to motility in this bacterium^9,32^. To experimentally determine the chemotaxis system that transduces the MCP_DRK_ signalling, we constructed mutants with complete deletions of the I/F9, II/F6 and III/F7 chemotaxis systems and analysed their response to d-Arg. Neither deletion of I/F9 nor of III/F7 reduced *V. cholerae’s* negative chemotactic response to d-Arg, strongly suggesting MCP_DRK_ association to the II/F6 chemosensory system (Extended Data Fig. 6a). Further evidence was obtained by investigating the localization of the MCP_DRK_ chemoreceptor in these mutant backgrounds. MCP_DRK_ foci overlap with those of CheY3 of the chemotaxis cluster II (Extended Data Fig. 6b) and are lost in the II/F6 but not in the III/F7 or I/F9 mutants, confirming that MCP_DRK_ belongs to the chemotaxis system II/F6 (Extended Data Fig. 6c).

### Structure of the sensory module of MCP_DRK_

**Fig. 3:**
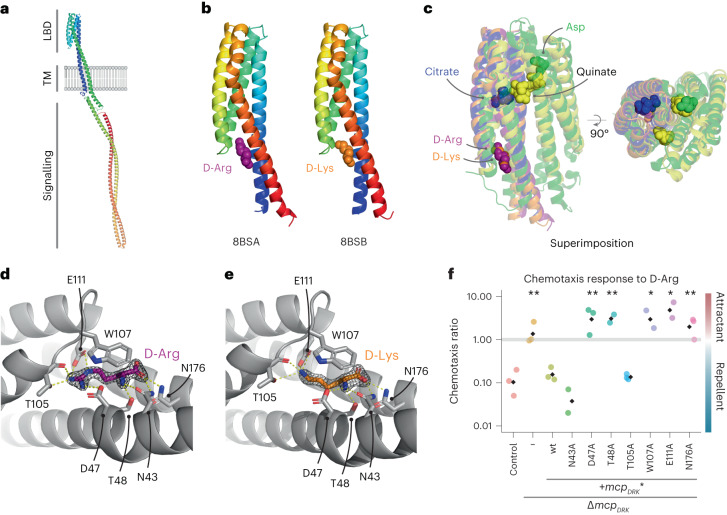
Structural basis of the MCP_DRK_ chemoreceptor binding d-Arg and d-Lys. **a**, Domain architecture of MCP_DRK_ protein generated by alphafold^40–42^, showing one subunit in rainbow colours, with the N terminus in blue and C terminus in red. The full-length MCP_DRK_ transmembrane chemoreceptor contains a ligand binding domain (LBD), a transmembrane (TM) and a signalling domain. **b**, MCP_DRK_-LBD monomers bound to d-Arg (purple spheres) or d-Lys (orange spheres) are coloured in rainbow colours from the N terminus (blue) to the C terminus (red). **c**, Superimposition of the LBD domain of MCP_DRK_ crystallized with d-Arg (in purple) and d-Lys (in orange) with the aspartate-bound Tar receptor from *E. coli* (PDB ID: 4z9i in green)^81^, the citrate bound MCP2201 chemoreceptor from *C. testosterone* (PDB ID: 5XUB, in blue)^34^ and the more promiscuous quinate-bound PcaY_PP chemoreceptor from *P. putida* (PDB ID: 6S38, in yellow)^35^. The Tar and the PcaY_PP receptor ligand binding sites are located at the dimer interface and are therefore shown as dimers. **d**, The binding site of MCP_DRK_-LBD bound to d-Arg (in purple). **e**, The binding site of MCP_DRK_-LBD bound to d-Lys (in orange). **f**, Functional analysis of the d-Arg binding pocket was performed by substituting residues Asn43, Asp47, Thr48, Thr105, W107, Glu111 and Asn176 with an alanine. Chemotactic response to d-Arg was then analysed for Δ*mcp*_*DKR*_ cells expressing either wild-type or mutant MCP_DRK_ under its native promoter. Δ*bsrV* strain was used as background. −, not complemented Δ*mcp*_*DRK*_. Black diamonds represent the mean of 3 independent biological replicates. Significant differences (unpaired *t*-test) are indicated by *P < 0.05 or **P < 0.01. Source data

MCP_DRK_ is a 59.1 kDa protein (Uniprot: Q9KSE4) that is predicted to have the typical transmembrane chemoreceptor domain architecture. MCP_DRK_ contains a ligand binding domain (LBD) predicted to belong to the 4HB_MCP protein family (Pfam PF12729) as a sensory module, a transmembrane HAMP domain (Pfam PF00672) and a cytoplasmic MCP-signalling domain (Pfam PF00015) as output module (Fig. 3a). To elucidate the molecular mechanisms of ligand recognition by MCP_DRK_, we purified the LBD domain of MCP_DRK_ (20 kDa) with a Glutathione S-transferase (GST) tag that was cleaved off before the final size-exclusion chromatography (SEC) step. This purified LBD was monomeric in solution, both in the absence and presence of its d-Arg ligand, as determined by multi-angle laser light scattering (SEC-MALS, see Supplementary Fig. 2). We solved two X-ray crystal structures of MCP_DRK_’s LBD in complex with either d-Arg or d-Lys at 1.8 Å resolution (PDB 8BSA and 8BSB, Supplementary Table 3). The crystals belonged to space group P1 and contained two LBD molecules, organized head-to-tail, in the asymmetric unit. This organization in the crystal is highly unlikely to be a physiologically relevant assembly since the membrane-proximal subdomain of one subunit packs against the membrane-distal subdomain of the other. As the monomers in the asymmetric unit were virtually identical and both were ligand bound, we here only show and discuss the A-chain of each structure. The final structure models correspond to residues 32–187 or 32–189 for the d-Arg and d-Lys-bound structures, respectively.

Overall, MCP_DRK_-LBD forms an antiparallel four-α-helix bundle structure (4HB) (Fig. 3b). This fold is distinct from the dCACHE LBD-type fold that is found in other chemoreceptors from *V. cholerae* that respond to l-amino acids (Mlp24, Mlp37) (refs. ^11,12^), but has been found in various LBDs from other Gram-negative bacteria. A structure-based homology search via the DALI server^33^ showed that MCP_DRK_-LBD was structurally most similar to the citrate-binding chemoreceptor MCP2201 from *Comamonas testosterone* (PDB ID: 5XUB, RMSD of 1.7 Å) (ref. ^34^) and the more promiscuous PcaY_PP chemoreceptor from *Pseudomonas putida* (PDB ID: 6S38, RMSD of 2.1 Å) (ref. ^35^), but also showed similarity to the well-characterized aspartate-binding Tar receptor from *E. coli* (PDB ID: 4Z9I, RMSD of 2.5 Å) (ref. ^36^). While these LBDs share a similar overall fold, their ligand binding sites are at entirely different locations (Fig. 3c). For instance, the model receptors Tar and Tsr bind ligands at the dimer interface with negative cooperativity^37–39^, while MCP_DRK_ binds d-Arg and d-Lys centrally and remains as monomer instead (Fig. 3c and Supplementary Fig. 3). As for MCP_DRK_, the chemoreceptor MCP2201 from *C. testosterone* also binds the ligand to a single protein chain, but at a different location (Fig. 3c)^34^.

In the MCP_DRK_-LBD structures, both d-Arg and d-Lys bind at the same site, at the bottom of the second and the third helix (Fig. 3b). To bind the ligands, Asp47 forms an ionic bond with the positively charged α-amine group of both DAAs. Further, Glu111 and Asp47 interact with the delocalized positive charge of the guanidino group from d-Arg; in a similar manner, the positively charged side-chain amine of d-Lys forms a salt bridge with Glu111. The ligands are further stabilized by hydrogen bonds with residues from all helices, namely Asn43, Thr48, Thr105, Glu111 and Asn176 (Fig. 3d,e). Trp107, from the short loop between helix 2 and 3, forms a lid that closes off the binding site and has a stacking interaction with the ligands.

To further characterize the precise mechanism of ligand specificity, we generated seven individual mutants in which each amino acid residue that interacts with the ligands according to the crystal structures was replaced with alanine (N43A, D47A, T48A, T105A, W107A, E111A and N176A). Complementation assays were performed by introducing these mutant alleles under the control of *mcp*_*DRK*_ native promoter interrupting the *lacZ* locus in the Δ*mcp*_*DRK*_ strain. Chemotaxis assays demonstrated the essentiality of 5 of the 7 MCP_DRK_-LBD residues tested (N43, T48, W107, E111 and N176) to promote a repulsive response to d-Arg in *V. cholerae* (Fig. 3f). d-Arg-mediated taxis was not affected by the N43A and T105A mutations, suggesting a less relevant role for these residues in stabilizing the d-Arg and d-Lys. These in vivo assays thus confirmed the location of the ligand binding site that was found in the crystal structure.

### Conservation of MCP_DRK_

**Fig. 4:**
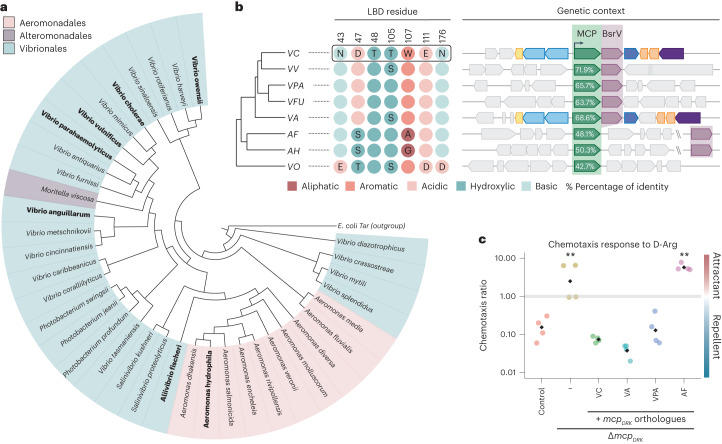
Specificity and conservation of MCP_DRK_. **a**, Tree of MCP_DRK_ orthologues. The cladogram was constructed on the basis of MCP_DRK_ sequence homology. Orthologues selected for an extended comparison are highlighted in bold. **b**, Left: selection of MCP_DRK_ orthologue representatives and the conservation of d-Arg binding residues. Only the residues that differ from the reference protein (VC1313, that is, MCP_DRK_ from *V. cholerae* El Tor strain N16961, boxed) are labelled. Coloured circles indicate the amino acid type (N, asparagine; D, aspartic acid; T, threonine; W, tryptophan; E, glutamic acid; S, serine; A, alanine; G, glycine). Right: genetic context of the chemoreceptor in several species. Both the MCP and the racemase are highlighted. The percentage of total protein identity (%) is shown. **c**, Chemotactic response of MCP_DRK_ orthologue proteins to d-Arg. The chemotactic response to d-Arg was tested for Δ*mcp*_*DKR*_ cells complemented with several orthologue MCPs expressed under the *mcp*_*DKR*_ native promoter. Δ*bsrV* strain was used as background. −, not complemented Δ*mcp*_*DRK*_; VC, *V. cholerae*; VV, *V. vulnificus*; VPA, *V. parahaemolyticus*; VFU, *V. furnissii*; VA, *V. anguillarum*; AF, *Aliivibrio fischeri*; AH, *Aeromonas hydrophyla*; VO, *V. owensii*. Black diamonds represent the mean of 4 independent biological replicates. Significant differences (unpaired *t*-test) are indicated by **P < 0.01. Source data

**Extended Data Fig. 7:**
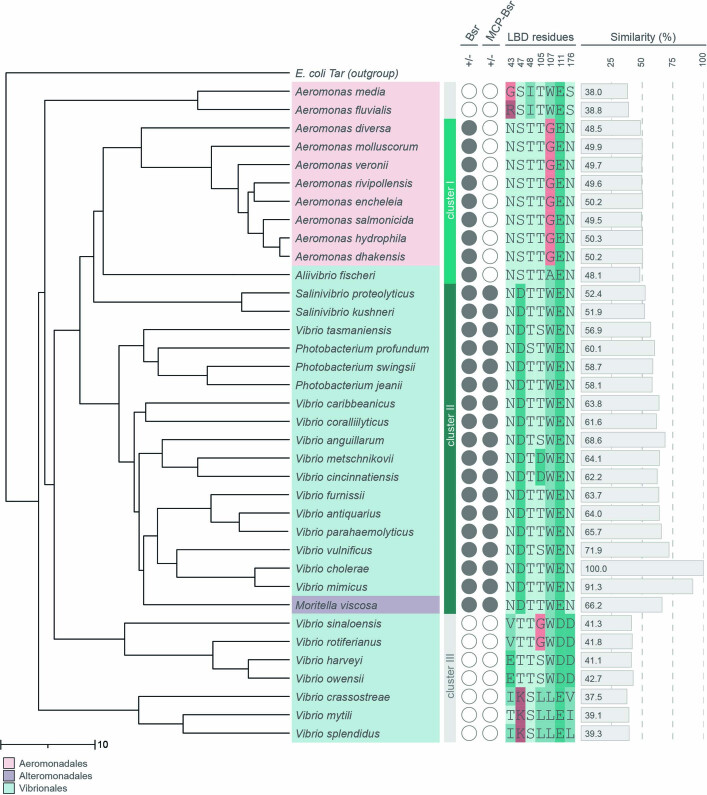
Tree of MCP_DRK_ orthologues. The cladogram was constructed based on MCP_DRK_ sequence homology. Representative species and strains were downloaded from Biocyc database. We included the non-related *E. coli* Tar chemoreceptor to help root the tree (light gray). In the first column, the presence of a Bsr ortholog is represented as full (presence) and empty (absence) circles. The presence of Bsr ortholog clustered together with the racemase is also represented in a similar way in the second column. Conservancy of the LBD residues implied in D-Arg binding is also shown (residue alignment). The bar chart represents the complete protein identity percentage (%). Bacteria were clustered in 3 different groups (cluster I, II, III) depending on the presence/absence of Bsr and MCP_DRK_ orthologs.

To analyse the conservation of MCP_DRK,_ we built a phylogenetic tree on the basis of sequence similarity (Fig. 4a). We found that this chemoreceptor is widely conserved in the Gamma-proteobacteria orders Aeromonadales, Alteromonadales and Vibrionales, which are known to be polar flagellated bacteria that are commonly found in aquatic environments. MCP_DRK_-like proteins cluster into three groups on the basis of their percentage of homology and genomic association with the BsrV racemase (Extended Data Fig. 7). MCP_DRK_ orthologues with the highest protein identity (>50%) cluster together with a BsrV-like racemase (for example, many *Vibrio* species and *Moritella* sp.), suggesting coevolution of d-amino acid production and chemotactic recognition. In these orthologues, the d-Arg/d-Lys binding site residues are either fully conserved or have only subtle changes (for example, *V. metschnikovii, V. cincinnatiensis* and *V. anguillarum*; see Extended Data Fig. 7). Species with MCP_DRK_ orthologues of slightly lower identity (48–51%) (for example, *Aeromonas* and *Aliivibrio*) form a single clade and typically show a substantial change in two important binding site residues: D > S in position 47, resulting in a loss of the salt bridge with the ligand, and W > G in position 107, which removes the stacking interaction with the ligand. Remarkably, in these species, the BsrV-like racemase does not form an operon with the chemoreceptor but is encoded elsewhere in the genome. Finally, the species encoding MCP_DRK_ homologues of lower identity (<45%) (for example, *V. owensii*) contain neither the d-Arg/d-Lys binding residues nor a BsrV-like locus.

To assess the potential functional divergency of the MCP_DRK_ chemoreceptor across species, we selected three orthologues from the pool of representative genomes that we used to build the phylogenetic tree. These were *V. anguillarum* VAA_1440 and *V. parahaemolyticus* VPA_1000, which cluster with a BsrV-like racemase and show a conserved binding site. We also selected *A. fischeri* VF_A1069, which does not form an operon with the racemase and has a partially conserved binding site (Fig. 4b). LBD model analysis on models generated by alphafold^40–42^ predicted defective d-Arg stabilization caused by changes in the VF_A1069 binding site as compared to the canonical one (that is, *V. cholerae*) (Supplementary Fig. 3). To study this, we monitored by capillary assays the response to d-Arg of the Δ*mcp*_*DRK*_ mutant strain complemented with the selected orthologues expressed under the *mcp*_*DRK*_ native promoter. As expected, we found that expression of VAA_1440 (68.6% identity, 100% conserved binding site) and VPA_1000 (65.7% identity, 100% conserved binding site) restored the ability of the Δ*mcp*_*DRK*_ mutant to sense d-Arg. The expression of VF_A1069 (48.1% identity, 71% conserved binding site) did not complement the chemotactic response to d-Arg (Fig. 4c), probably because this orthologue does not conserve the D47 and W107 residues (Supplementary Fig. 3) that were found to be essential for d-Arg binding (Fig. 3). Together, these results suggest that MCP_DRK_-like proteins specific for d-Arg and d-Lys are those encoded nearby a *bsr* locus.

## Discussion

Bacterial chemotaxis has been the subject of decades of research. Although the signal transduction components have been extensively investigated, less is known about the identity and specificity of the chemoreceptors that bacteria use to sense their surroundings^43^. Given the intimate association between MCPs and a bacterium’s lifestyle, these chemoreceptors are generally numerous and specific, suggesting that many MCP–ligand pairs remain uncharacterized^6^.

We previously reported that many bacteria release high concentrations of a variety of d-amino acids to the environment. d-amino acids display different biological functions depending on their chemical properties. For instance, stationary-phase *V. cholerae* cells produce d-Met to adapt their cell wall biogenesis to growth arrest mode^17^ while they use d-Arg to inhibit the growth of nearby competitors^15^. While d-Arg is lethal to many species, *V. cholerae’s* physiology appears to be unaffected by this d-amino acid^15^. Using a mutant defective in d-amino acid production, we report here that d-Arg is sensed by a previously unrecognized MCP to move the *V. cholerae* population away from stressful environments.

**Fig. 5:**
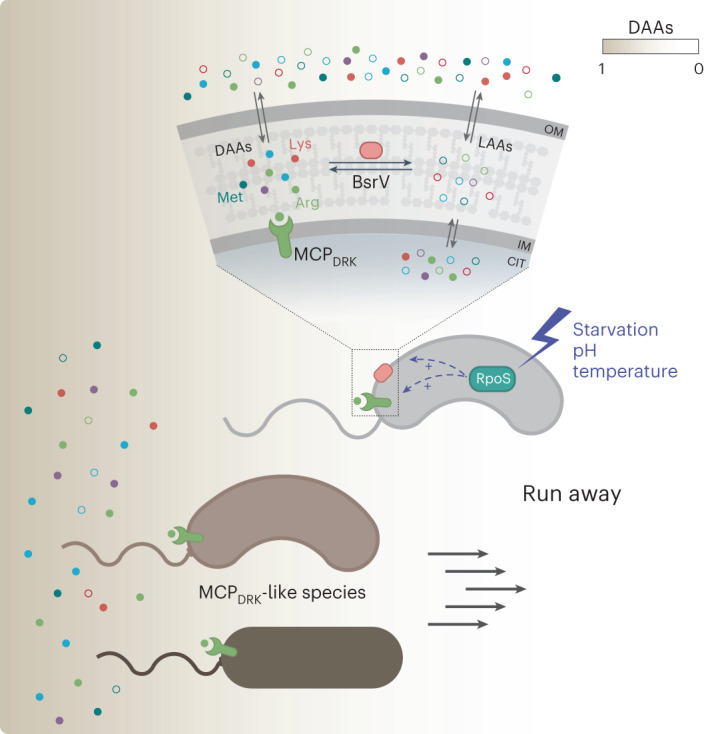
Model of MCP_DRK_-dependent chemotactic response to d-amino acids. Under certain environmental stresses (that is, starvation), the RpoS response induces the expression of both the broad-spectrum racemase BsrV and the dedicated d-Arg/d-Lys chemoreceptor MCP_DRK_. Among the d-amino acids (DAAs) produced by BsrV, d-Arg and d-Lys stand out as warning signals sensed by *V. cholerae* as well as other MCP_DRK_-encoding species. Chemotactic run-away response to these d-amino acids enables these bacterial communities to move away and explore more favourable niches.

It is interesting that among all the d-amino acids produced by *V. cholerae*, only the toxic d-Arg (and d-Lys) induce negative chemotaxis. This means that d-Arg plays multifaceted roles in shaping the biodiversity and structure of microbial communities when nutrients are scarce by clearing the environment of potential competitors while also migrating the community towards more favourable niches. For this ‘fight and flight’ strategy, *V. cholerae* has evolved to arrange the genes encoding the d-Arg-sensing MCP (MCP_DRK_) and the broad-spectrum racemase (BsrV) together in one operon whose expression is controlled by the stress sigma factor RpoS. By synchronizing the production of d-Arg and its MCP, *V. cholerae* builds an efficient stress-dependent response while simultaneously preventing its futile activation under favourable conditions (Fig. 5). In fact, when the MCP_DRK_ is not expressed, d-Arg stimulates a mild chemotactic attraction that suggests the existence of other MCPs with lower affinity for this ligand. Indeed, our TPP experiments identified potential MCP candidates whose stability is affected by d-Arg, such as VC2161 (Mlp24) and VC1406 (Fig. 1f). Chemotaxis towards d-Arg may have a nutritional purpose. This idea is supported by results in *E. coli* showing that certain compounds with low nutritional value can be chemoattractants^44^ and by the presence of a catabolic d-amino acid dehydrogenase enzyme^45^.

The crystal structures of the LBD of MCP_DRK_ showed that it has a typical antiparallel 4HB structure, which is conserved in various receptors^46^. Interestingly, this relatively simple fold has evolved to recognize a variety of substrates by using different binding sites. The binding site of Tar, Tsr and the PcaY_PP receptors is located at the dimer interface, while MCP_DRK_ and MCP2201 bind their ligands centrally within the 4HB (Fig. 3c). MCP_DRK_’s d-Arg-binding pocket is located at the bottom of the second and the third helix, where only five residues are essential for recognizing this d-amino acid and similar compounds such as d-Lys. Contrary to the 4HB-containing model receptors, that is, Tar and Tsr, our purified MCP_DRK_-LBD construct does not oligomerize in the presence of the ligand. This is also in contrast with MCP2201, where ligand binding inside the monomer induces a structural rearrangement that prevents dimerization of the LBD, instead stimulating trimerization^34^.

Interestingly, although MCP_DRK_ is broadly conserved among species from three Gamma-proteobacteria orders, the specificity for d-Arg/Lys seems to be evolutionarily restricted to receptors that are transcriptionally linked to broad-spectrum racemases such as BsrV. As some of these species are both tolerant to d-Arg toxicity^15^ and inhabit aquatic habitats, d-Arg/d-Lys produced by one of these species could simultaneously coordinate the chemotactic response for many inhabitants of the same niche. Conversely, species that lack a Bsr homologue or encode it somewhere else in the genome display changes in the ligand binding domain that are likely to disable the chemotactic response to d-Arg/d-Lys (Fig. 4 and Extended Data Fig. 7). While some of these changes imply critical structural variations in the binding site (Supplementary Fig. 3), more subtle ones could instead recognize alternative signals that are chemically related to d-Arg. Indeed, multiple arginine analogues are known to be naturally produced, such as l-homoarginine in humans and animals^47^ and l-canavanine and l-indospicine produced by certain plants^48^. Interestingly, canavanine can be racemized by microbial Bsr enzymes, suggesting that it should be possible to find both l- and d-enantiomers of arginine analogues in the natural environment^49^. Future studies should investigate how interspecies and interkingdom competing signals for the same MCP may impact chemotaxis regulation and hence bacterial capacity to respond to stress in complex polymicrobial environments.

The role of d-amino acids as chemotactic repellent signals adds to previously reported roles for these molecules in stationary-phase adaptation. An obvious ecological scenario where the d-Arg run-away response could be beneficial is abandoning spent environments. It is known that *V. cholerae* forms biofilms during the aquatic and intestinal phases of its life cycle^50^; when the biofilm ages, RpoS controls biofilm detachment^51^. In a host environment, RpoS-dependent activation of chemotaxis and motility is linked to mucosal escape. Given that this response coincides with a reduction in the expression of virulence genes, it has been proposed as a preparation of *V. cholerae* for the next stage in its life cycle^52^. While several degradative proteins contributing to biofilm disassembly have been reported in recent years (for example, in *Vibrio*^25^), the signal(s) promoting biofilm dispersal remain enigmatic. Therefore, an exciting question for the future will be whether d-Arg’s dual function as a broad-spectrum toxin and chemotactic warning signal could play a role in biofilm destablization and dispersal. In this line, a study recently reported that the MCP_DRK_-BsrV operon (*vc1313-1312*) is part of the in-biofilm *repressome* and that constitutive expression of some of these genes interferes with biofilm formation^53^. However, the loss of MCP_DRK_ was reported to have no effect on *V. cholerae’s* fitness in infection or dissemination^54^. While the effect of d-Arg chemotaxis on virulence and transmission remains to be studied, the widespread presence of this chemoreceptor in other marine non-pathogenic bacteria suggests that this response could be an important and overlooked aspect of bacteria’s ability to assess niche habitability.

## Methods

### Bacterial growth conditions

Strains are summarized in Supplementary Table 4. All *V. cholerae* strains are derivatives of the sequenced N16961, an El Tor clinical isolate^55^.

Bacteria were grown using standard laboratory conditions. Cultures were streaked onto LB plates (1% tryptone, 0.5% yeast extract, 1% NaCl, 15% bacto-agar) supplemented with antibiotics or amino acids (see below), and single colonies were selected and grown overnight at 30 °C in tryptone broth (TB) (1% tryptone, 0.5% NaCl) and subcultured in fresh TB until the desired growth phase, unless otherwise specified. Antibiotics were added when necessary at the following concentrations: 200 μg ml^−1^ streptomycin (Sm, Duchefa Biochem, S0148), 100 μg ml^−1^ carbenicillin (Cb, Duchefa Biochem, C0109) and 50 μg ml^−1^ kanamycin (Kn, Duchefa Biochem, K0126). Soft-agar plates were supplemented with 5 mM amino acids (Sigma). Optical density (OD) was measured in cuvettes using an Ultrospec 7000 spectrophotometer (GE Healthcare). For experiments in minimal medium, *V. cholerae* was grown in either M9 minimal medium supplemented with 1 μg ml^−1^ thiamine or MSR6 (50 mM KH_2_PO_4_, 50 mM Na_2_HPO_4_, 7.5 mM (NH_4_)_2_SO_4_, 2 mM MgSO_4_, 0.1 mM CaCl_2_, 25 μM FeSO_4_, 0.04% w/v d-glucose and 0.4% w/v succinate).

For growth curves, stationary-phase cultures were normalized to an OD of 0.04 and 20 μl were used for inoculating 96-well plates containing 180 μl of fresh medium. At least 3 replicates per strain and condition were inoculated in 2 independent experiments. Optical density was monitored using Biotek Gen5 (v.08) in an Eon Biotek plate reader (Biotek) at 5 min intervals at 30 °C.

### Construction of mutants and plasmids

All mutants were generated by in vivo allelic exchange as previously described^56^. In brief, 1 Kb flanking regions were cloned into XbaI-digested pCVD442 to generate recombinant suicide vectors. For conjugations into *V. cholerae*, stationary-phase recipients and Sm10 λpir donor strains were washed of antibiotics, mixed in equal ratios and spotted onto LB without selection. After 4–6 h incubation at 37 °C, cells were streaked onto Cb/Sm-supplemented LB plates to select for transconjugants. Finally, cells were cured through a counterselection round on salt-free 10% sucrose plates. The chromosomal fluorescent *sfGFP*-*vc1313* reporter was constructed in a similar way, directly inserting *sfGFP* upstream of *vc1313* in-frame.

Plasmids and oligonucleotides used are summarized in Supplementary Tables 5 and 6, respectively. Plasmids were constructed by standard DNA cloning techniques. *bsrV* was PCR amplified using the appropriate primers including a strong ribosomal binding site (RBS) and cloned into restriction-digested pXB300 by isothermal assembly, yielding plasmid p*bsrV*. Similarly, for the generation of the phosphomimetic non-chemotactic mutant, *cheY3* was PCR amplified with primers carrying the desired substitutions (CheY3^D16K, Y109K^), and the fragment was isothermally assembled into restriction-digested pXB300 to generate pcheY3**. Plasmids p*bsrV* and pcheY3** were introduced into Δ*bsrV* and Δ*cheY3*, respectively, via electroporation.

The DNA fragment encoding the entire periplasmic domain (residues Ser32–Ser191) of *vc1313* (herein referred to as *vc1313-LBD*) was PCR amplified and subcloned into BamHI/EcoRI-digested expression vector pGEX-6P-2 by isothermal assembly so that the N-terminal GST-encoding sequence was fused in-frame to yield pGEX-*vc1313-LBD*. The stop codon located downstream of the multicloning region was used, therefore the resulting protein contained an additional C-terminal 12-amino acid arginine-rich tail.

Δ*vc1313* complementation was achieved by expressing a copy of *vc1313* under the control of its native promoter introduced in neutral locus *lacZ*. *vc1313* fragment was PCR amplified (including 500 bp upstream of the promoter region) and isothermal assembled into restriction-digested pJL1, a suicide plasmid that permits allele insertion into *V. cholerae lacZ*^57^. Successful integration was first verified by blue–white screening on plates containing 5-bromo-4-chloro-3-indolyl-β-d-galactopyranoside (X-Gal, 40 μg ml^−1^, Sigma, B4252) and later checked by PCR. Site-directed mutagenesis of the *vc1313* gene was carried out following Q5 site-directed mutagenesis protocol (NEB) using pJL1::*vc1313* plasmid as template.

The fidelity of all generated mutants and plasmids was confirmed by DNA sequencing.

### Soft-agar motility assay

Single colonies of *V. cholerae* strains were grown overnight in LB broth at 30 °C with shaking, subcultured 1:100 in fresh broth and grown until exponential phase. A 2 μl volume of the normalized cultures were then spotted into 0.3% soft-agar plates and drops were allowed to dry at room temperature. When indicated, plates were supplemented with 5 mM of amino acids. After inoculation, plates were incubated for 8 h at 30 °C and then imaged in a LAS-3000 imaging system (Fuji). Image analysis and processing were performed using Fiji/ImageJ^58^ and results were plotted using GraphPad Prism (v.8.0).

### d-amino acids quantification

Total d-amino acid concentration was determined by a colorimetric d-amino acid oxidase (DAAO) assay adapted to a 96-well plate format as previously described^14,15^. Briefly, 20 µl of sample were mixed with 60 µl of reaction buffer (sodium phosphate buffer 33 mM, pH 7.5, *Trigonopsis variabilis* DAAO (ref. ^59^) 0.04 mg ml^−1^, horseradish peroxidase 0.04 mg ml^−1^, o-phenylenediamine 83 ng ml^−1^ and flavin adenine dinucleotide (FAD) 8.3 ng ml^−1^) and incubated for 1 h at 37 °C. The reactions were inactivated by adding 160 μl 2 M HCl and measurements were taken at 292 nm in an Eon Biotek plate reader (Biotek).

### Capillary assays

Chemotactic ability was examined using previously described capillary assays^60^, with some modifications. An overnight culture of *V. cholerae* was diluted 1:200 into fresh M9 minimal medium and incubated until late exponential phase at 30 °C with shaking. Cells were collected by low-speed centrifugation, washed twice with motility buffer (MB) (50 mM HEPES (pH 7.4), 300 mM NaCl, 10 mM glucose, 5 mM MgCl_2_) and finally resuspended to a final OD of 0.1 in MB. Tween20 was added to a final concentration of 0.01% to avoid cell adhesion. After preincubation of cells at r.t. for 1 h, pre-filled 1 µl microcapillary tubes (Sigma, P1421) containing the amino acids of interest (500 µM, unless specified otherwise) were placed in contact with the cell suspension and the suspension was incubated at r.t. for an additional 2 h. Finally, the number of bacteria (colony-forming units per ml) in each capillary was counted by plating serial dilutions on LB agar, and chemotaxis ratio was calculated relative to a control capillary.

### Microfluidic chemotaxis experiments

Microfluidics chambers were fabricated as previously described^29^. Briefly, the SU8-based negative master mold of the microfluidic devices was produced by standard photolithography techniques. Microfluidics devices were produced from the mold using polydimethylsiloxane (1:10 crosslinker-to-base ratio, thermal-cured overnight at 65 °C), which was cut to shape and bound to glass slides via oxygen plasma treatment. After production, devices were stored short-term in sterile deionised water to retain their hydrophilic properties. *V. cholerae* strains were grown overnight in TB at 30 °C from glycerol stocks, diluted 1:100 in 10 ml M9 medium and grown for 4.5 h at 30 °C with shaking until an OD of ~0.3. The day culture was washed three times in motility buffer and diluted to a final OD of 0.1. The microfluidic device consists of two large chambers linked by a small straight channel (length = 2 mm, width = 1 mm, height = 70 µm). The suspension of cells at OD = 0.1 was introduced in one chamber, while the second was filled with MB supplemented with indicated amounts of the tested compound. The device’s inputs were then sealed with microscopy grease to prevent evaporative flows. A gradient of the compound forms rapidly (~1 h) in the connecting channel. The resulting chemotactic motion of the cells was measured in the centre of the channel 1, 2 and 3 h after loading the device. To this end, the sample was observed under phase contrast microscopy at ×10 magnification (NA 0.3) and a 10,000-frames movie was recorded at a rate of 200 frames per second using a Mikrotron Eosens 4CXP CMOS camera with a 717 × 717 µm² (512 × 512 px²) field of view. A control with the same cell suspension but only MB in the second chamber was always performed simultaneously.

### Swimming speed experiments

*V. cholerae* strains were grown overnight in TB (supplemented with 500 µM d-Arg if indicated) at 30 °C from glycerol stocks, diluted 1:200 in 20 ml TB (+500 µM d-Arg if indicated) and grown for 3 h at 30 °C with shaking until an OD of ~0.6. The cells were then washed into fresh TB. A small volume (3 µl) of the suspension of cells was sandwiched between two coverslips maintained 100–200 µm apart by microscopy grease. Cell motion was recorded at mid-height between the coverslips using the same microscopy protocol as described above.

### Swimming speed and chemotactic bias measurement

Movies were analysed offline using the differential dynamic microscopy (DDM) and phase differential microscopy (PhiDM) methods implemented as plugins in ImageJ (https://github.com/croelmiyn/FourierImageAnalysis) to measure swimming speed^61^ and chemotactic drift^62^, respectively. The DDM algorithm computes the time autocorrelation of the spatial Fourier components of the images, which are then fitted with a model assuming that a fraction of the cells is non-motile and diffusing, and the other is motile, with swimming speeds being Schulz distributed around a mean. Consistent fits across the different Fourier components yield the fraction of swimming cells $\Phi$ and their average swimming speed ${v}_{0}$. With PhiDM, the temporal shift in the phase of the same Fourier components is fitted to measure the population-averaged drift velocity of the cells in the field of view, ${v}_{d}$. In the absence of external flows, this drift is solely due to the chemotactic motion of the motile cells since the non-motile cells do not drift on average. The chemotactic drift velocity of the motile cells was therefore computed as ${v}_{{\mathrm{ch}}}={v}_{d}/\Phi$. Since the chemotactic drift velocity is expected to scale with the swimming speed of the cells, we also computed the chemotactic bias $b={v}_{{\mathrm{ch}}}/{v}_{0}={v}_{d}/\Phi {v}_{0}$, which must lie in the range (−1, 1) and estimates the angular motion bias of the cells up (*b* > 0) or down (*b* < 0) the gradient.

### Thermal proteome profiling

*V. cholerae* cells were grown overnight at 37 °C in LB and diluted 100-fold into 100 ml of fresh LB. Cultures were grown aerobically at 37 °C with shaking until an OD of ~2. Cells were pelleted at 4,000 × *g* for 5 min, washed with 10 ml PBS and resuspended in 1.2 ml of lysis buffer (final concentration: 50 μg ml^−1^ lysozyme, 250 U ml^−1^ benzonase and 1 mM MgCl_2_ in PBS). Cells were lysed by 5 repeated cycles of freeze–thawing (freezing in liquid nitrogen, followed by 5 min at 25 °C with shaking until complete thawing). Then, d-Arg was added at 4 different concentrations (0, 0.05, 0.4, 3.2 mM) and 20 μl was aliquoted to a PCR plate and immediately subjected to a temperature gradient for 3 min in a PCR machine (Agilent SureCycler 8800), followed by 3 min at room temperature. NP40 was then added to all conditions to a final concentration of 0.8%. The plate was then centrifuged at 2,000 × *g* for 5 min to remove cell debris and the supernatant was filtered at 500 × *g* for 5 min through a 0.45 μm 96-well filter plate (Millipore, MSHVN4550) to remove protein aggregates. The flow-through was mixed 1:1 with 2× sample buffer (180 mM Tris (pH 6.8), 4% SDS, 20% glycerol, 0.1 g bromophenol blue) and kept at −20 °C until analysis. Mass spectrometry-based proteomics (using tandem mass tags) was then used to quantify the amount of protein in each condition, as previously described^63^. Data were analysed by a method to detect dose-dependent changes in protein thermal stability^27^.

### Western blotting to determine presence of vc1313

An overnight culture of *V. cholerae* sfGFP-*vc1313* was diluted 1:100 into fresh LB minimal medium and incubated at 37 °C with shaking. OD_600_ was measured and samples were taken as a function of time. Samples were then normalized to total protein amount and analysed by SDS–PAGE. Western blotting was performed using specific antibodies against GFP-tag (1:500, Thermo Fisher, A-11122). GFP signal was detected using Amersham Imager 600 (GE) and analysed using Fiji/ImageJ (v.1.53f51) (ref. ^58^).

### Time-lapse microscopy

Bacterial cells were immobilized onto agarose pads (1% agarose w/v, 20% v/v PBS and 20% v/v LB) on microscope slides. Phase contrast microscopy was performed using a Zeiss Axio Imager.Z2 microscope (Zeiss) equipped with a Plan-Apochromat ×63 phase contrast objective lens and an ORCA-Flash 4.0 LT digital CMOS camera (Hamamatsu Photonics) using the Zeiss Zen 2 Blue edition (v2.0.0.0) software. Image analysis and processing were performed using Fiji/ImageJ (v.1.53) (ref. ^58^) and MicrobeJ plugin (v.5.13) (ref. ^64^).

To follow the detailed localization of sfGFP-MCP_DRK_ during growth, cells were added into 50 ml of TB medium and growth was followed by measuring optical density. For each datapoint, samples were analysed by fluorescence microscopy and the percentage of cells with sfGFP-MCP_DRK_ foci was counted. For the carbon catabolite repression assays, cells were grown in MSR6 medium supplemented with 0.04% w/v d-glucose and 0.4% w/v succinate, and a similar procedure was followed.

### Demographic analysis of microscopy data

Demographic analysis was performed in several steps. Fluorescence intensity profiles of sfGFP-VC1313 cells were first measured using Fiji/ImageJ (v.1.53f51) (ref. ^58^). The generated data were then processed using the cellProfiles package (v.3.0.1; developed by Todd Cameron) in RStudio (v.1.4.1106; www.rstudio.com) to sort cells by length and normalize the generated intensity profiles as an average of each cell’s fluorescence. Finally, the ggplot2 package was used to plot the demographics (v.3.3.5) (ref. ^65^).

### Expression and purification of MCP_DRK_-LBD

*E. coli* strain BL21 (DE3) (Novagen) carrying pGEX-*vc1313*-LBD was cultured in Terrific Broth (24 g l^−1^ yeast extract, 20 g l^−1^ tryptone, 4 ml l^−1^ glycerol, 0.017 M KH_2_PO_4_, 0.072 M K_2_HPO_4_) containing 50 μg ml^−1^ carbenicillin at 30 °C until cell density reached an OD_600_ of ~1. Isopropyl β-d-1-thiogalactopyranoside (Sigma, I6758) was then added to a final concentration of 0.1 mM to induce protein expression, and the culture was incubated for an additional 16 h at 18 °C. Cells were collected by centrifugation, suspended in 1x PBS (140 mM NaCl, 2.7 mM KCl, 10 mM Na_2_HPO_4_ and 1.8 mM KH_2_PO_4_ at pH 7.3) and disrupted using a pressure cell homogenizer FC600 (Julabo) followed by centrifugation at 70,000 × *g* at 4 °C for 1 h. After removing cell debris, VC1313-LBD was purified from the soluble fraction by affinity chromatography using Glutathione Sepharose 4B resin (Cytiva) previously equilibrated with PBS. Following 3 washing steps with cleavage buffer (50 mM Tris-HCl, 150 mM NaCl, 1 mM EDTA, 1 mM dithiothreitol (pH 7)), GST-VC1313 was eluted with 50 mM Tris-HCl (pH 8.0) buffer containing 10 mM reduced glutathione. Cleavage buffer was added to a final 1x concentration and the N-terminal GST tag was then cleaved using PreScission Protease (Cytiva) following manufacturer instructions (16 h at 4 °C). The resulting sample was then dialysed in 2 l dialysis buffer (50 mM Tris-HCl (pH 7.0), 150 mM NaCl, 1 mM dithiothreitol and 1 mM EDTA) at 4 °C for 16 h using Slide-A-Lyzer dialysis cassettes, 7K MWCO (ThermoFisher,66370). Afterwards, the protein mixture was incubated with Glutathione Sepharose 4B resin to remove cleaved GST, PreScission protease and the remaining GST-tagged VC1313. The flow-through was loaded on a Superdex 200 Increase 10/300 GL column (Cytiva) in 10 mM Tris-HCl and 150 mM NaCl (pH7.4). The purity of MCP_DRK_-LBD was analysed by SDS–PAGE.

### Crystallization and structure determination

Purified MCP_DRK_-LBD was loaded on a Superdex 200 Increase 10/300 GL (Cytiva) equilibrated in 10 mM Tris and 150 mM NaCl (pH 7.4). Protein peak fractions were concentrated to 20–30 mg ml^−1^ on a 10 kDa cut-off Amicon Ultra centrifugal filter (Merck–Millipore).

Before setting up the drops, 13 mg ml^−1^ MCP_DRK_-LBD was mixed with either 10 mM d-arginine or 10 mM d-lysine, both from a 200 mM stock. The protein–ligand mixtures were mixed with reservoir solution at a 3:1 ratio. All crystals were grown at 20 °C by sitting-drop vapour diffusion in conditions containing 0.1 M Tris (base) bicine (pH 8.6–8.8), 17–21% v/v poly(ethylene glycol) methyl ether 500 (PEG 500 MME) and 8–10% w/v PEG 20000. Before flash freezing the crystal in liquid nitrogen, the PEG concentrations were raised to 23% v/v PEG 500 MME and 12% w/v PEG 20000.

X-ray diffraction data of the different crystals were collected on the beamlines ID30B and ID23-2 at the European Synchrotron Radiation Facility in Grenoble, France^66^. The data were processed using XDS^67^. The d-lysine and d-arginine crystals both belonged to the P1 space group and contained 2 molecules in the asymmetric unit. The crystallographic phase problem of the MCP_DRK_-LBD bound to d-arginine was solved by Arcimboldo^68^ using 8 helices of 14 residues as a search model. The structures of MCP_DRK_-LBD bound to d-lysine were solved by PHASER^69^ using the MCP_DRK_-LBD:d-Arg structure as a molecular replacement model. Coot (0.9.5) (ref. ^70^) was used to build the models and the structures were refined using Refmac5 (v.5.8.0267) (ref. ^71^) and PHENIX refine (v.1.13) (ref. ^72^). For complete data collection and refinement statistics, see Supplementary Table 3. The final models of the MCP_DRK_-LBD structures were validated using MolProbity^73^. Atomic coordinates and structure factors of the MCP_DRK_-LBD crystal structures (in complex with d-Arg or d-Lys) have been deposited in the Protein Data Bank (PDB codes 8BSA and 8BSB, respectively).

### Phylogenetic analysis

Nucleotide and amino acid sequences were obtained from the National Center for Biotechnology Information (NCBI)^74^ and the Biocyc database^75^. MCP_DRK_ homologues were identified using the BlastP algorithm^76^ against a non-redundant protein database, with an *E*-value threshold of 10^−10^ and with *V. cholerae* VC1313 as reference sequence. The MUSCLE algorithm^77^ was used to generate the multiple sequence alignment. The resulting alignment was then filtered by removing 90% of redundancy, and unique representative species were selected for further analysis. The Jalview (v.2.11.2.0) toolkit^78^ was used to visualize all the alignments and analyse the conservancy of residues of interest. The phylogenetic tree was generated using ClustalW2 (ref. ^79^), and Interactive Tree of Life (iTOL, v.5) (ref. ^80^) was then used to further customize the phylogenetic tree and add additional information. The genetic context analysis was carried out by comparing upstream/downstream regions of the selected representatives.

### Reporting summary

Further information on research design is available in the Nature Portfolio Reporting Summary linked to this article.

## Supplementary information

Supplementary Information Supplementary Figs. 1–3, Methods and Source Data.

Reporting Summary

Supplementary Tables 1–6 Supplementary Table 1. Thermal protein profiling results. The complete thermal protein profiling dataset is available in ProteomeXchange Consortium under identifier PXD038312. Table 2. Thermal protein profiling statistical analysis. Table 3. Crystallization data collection and refinement statistics. Table 4. List of strains. Table 5. List of plasmids. Table 6. List of oligonucleotides.

## Data Availability

The mass spectrometry proteomics data have been deposited to the ProteomeXchange Consortium via the PRIDE partner repository with the dataset identifier PXD038312. Atomic coordinates and structure factors of the MCP_DRK_-LBD crystal structures (in complex with d-Arg (PDB: 8BSA) and d-Lys (PDB:8BSB)) have been deposited in the Protein Data Bank. Source data are provided with this paper.

## Source data

Source Data Fig. 1 Statistical source data.
Source Data Fig. 2 Unprocessed western blot for Fig. 2b.
Source Data Fig. 2 Statistical source data.
Source Data Fig. 3 Statistical source data.
Source Data Fig. 4 Statistical source data.
Source Data Extended Data Fig. 1 Statistical source data.
Source Data Extended Data Fig. 2 Statistical source data.
Source Data Extended Data Fig. 4 Statistical source data.
Source Data Extended Data Fig. 6 Statistical source data.
