# Supplementary material for: d-amino acids signal a stress-dependent run-away response in Vibrio cholerae
Source: Nat Microbiol. 2023 Jun 26;8(8):1549–60. doi: 10.1038/s41564-023-01419-6 (PMC10390336; doi:10.1038/s41564-023-01419-6)
Supplement: Supplementary file 1 — Supplementary Figs. 1–3, Methods and Source Data. [file 41564_2023_1419_MOESM1_ESM.pdf]

nature microbiology

Article

https://doi.org/10.1038/s41564-023-01419-6

D-amino acids signal a stress-dependent run-away response in *Vibrio cholerae*

In the format provided by the
authors and unedited

Supplementary Information

Table of content

- Supplementary Figures 1-3
- Supplementary Methods
- Supplementary Source Data
- Supplementary References

Supplementary Figures

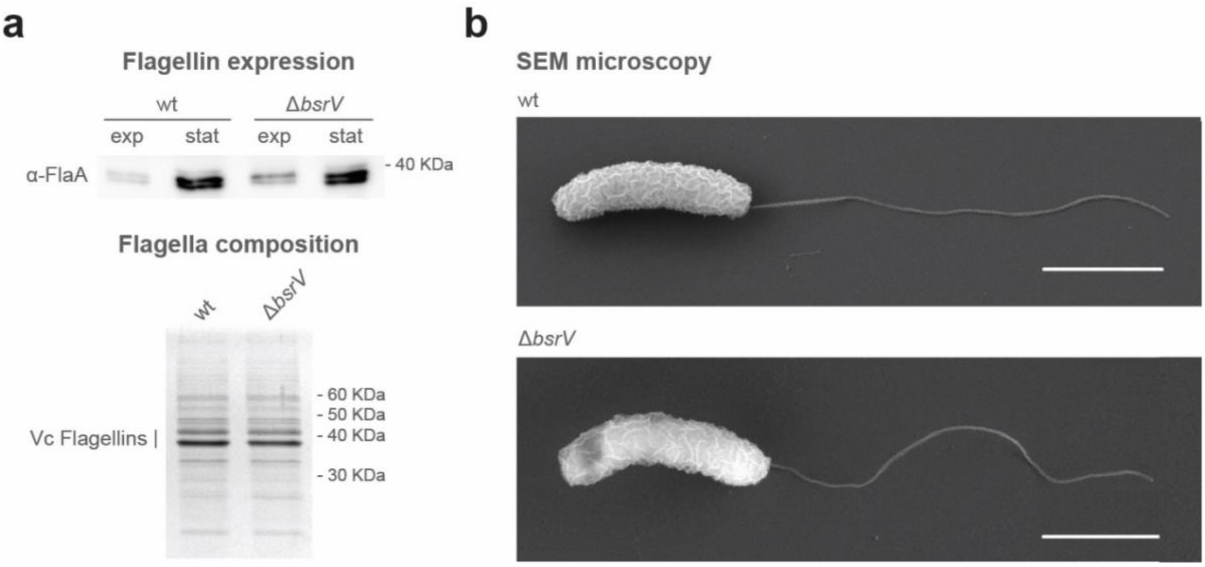

Supplementary Figure 1. Integrity of *V. cholerae* flagella. a, Analysis of the flagellin expression and composition *of V. cholerae* wild-type (wt) and Δ*bsrV* mutant cells. Western blot against *V. cholerae* flagellins using anti-FlaA (major flagellin) specific antibody at different growth phases is shown. Samples were normalized and loaded with equal total protein amount. exp: exponential growth phase. stat: stationary growth phase. The composition of the sheathed flagella was analysed by flagella purification followed by SDS-PAGE and Bradford staining. *V. cholerae* flagellins range between 39-41 KDa. The data shown are from a single experiment. **b**, Structural analysis of both strains by SEM at 50kX magnification. Representative micrographs are shown, at least 20 images were acquired per strain: Scale bar, 1 μm.

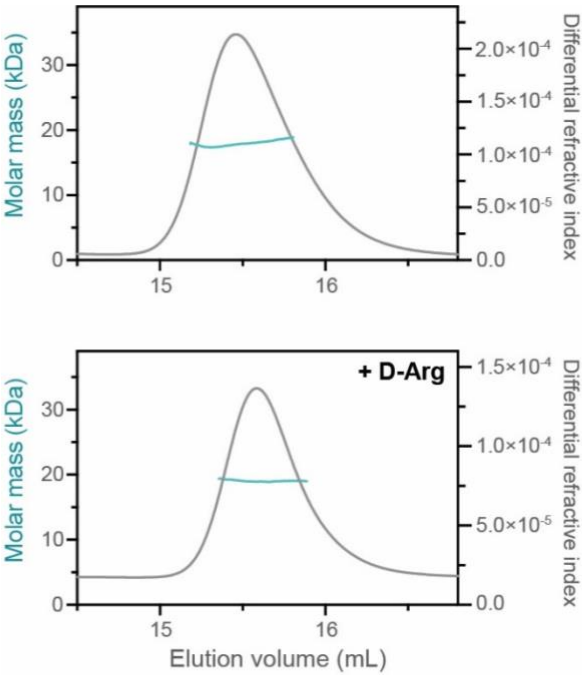

Supplementary Figure 2. SEC-MALs analysis. The chromatograms of size-exclusion chromatography are shown with or without D-Arg. The grey trace is the differential signal from the retractive index detector. The calculated mass of the protein is shown in tradewind-blue and is determined to be 18.0±0.1 kDa in absence and 19.2±0.2 kDa in presence of D-Arg (mean and standard deviation of 3 replicates). In both cases, MCP_{DRK}-LBD is a monomer in solution.

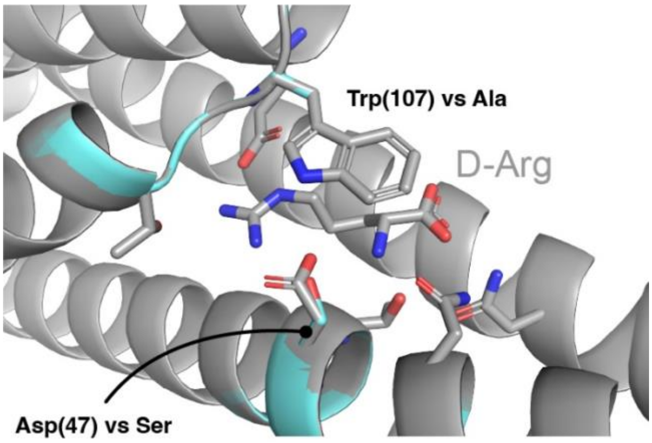

Supplementary Figure 3. Modelled binding sites for MCP_{DRK}-like chemoreceptors. Superimposed structures of *V. cholerae* MCP_{DRK} (in grey) and *A. fischeri* VF_A1069 alphafold model (in blue) chemoreceptor binding pockets. Residues that vary in VF_A1069 are highlighted.

Supplementary Methods

Western blotting to determine presence of flagellins

Overnight cultures of *V. cholerae* wild-type or *bsrV* mutant were diluted 1:100 into fresh LB minimal medium and incubated at 37 °C, shaking until the desired OD_{600}. Samples were then normalised to total protein amount and analysed by SDS-PAGE. Western blotting was performed using specific antibodies against FlaA. Primary antibody rabbit anti-FlaA (1:2000, in-house produced) and secondary antibody anti-rabbit-HRP (1:20000, Sigma, A0545-1ML) were used, and the chemiluminescent signal was detected by Amersham Imager 600 (GE) and analysed by Fiji/ImageJ [version 1.53f51][1].

Flagellin composition analysis

V. cholerae cells were grown overnight at 37 °C in LB, diluted 1:100 into 100 ml of fresh LB and incubated at 37 °C until late-exponential phase. Cells were harvested by centrifugation at 3,000 × g for 15 min and resuspended in 0.5M Tris-HCl pH8. 2-mm glass beads were then added to the culture and the samples were vortexed to separate the flagella from the body. Supernatant was recovered and clarified by ultracentrifugation 100,000 × g for 90 minutes. The final pellet was resuspended in PBS 1x. A fraction of the sample was boiled for 10 minutes and flagellin composition was analysed by SDS-PAGE.

SEM imaging

For flagella morphology analysis, *V. cholerae* cells were grown in TB until late-exponential phase and harvested by low-speed centrifuging at 5000 x g, for 5 min. After two washing steps in PBS1x cells were fixed in 2.5% glutaraldehyde in 0.1 M sodium cacodylate buffer for 16 hours at 4 °C. The day after, fixed cells were washed two additional times with 0.1 M sodium cacodylate buffer and dispersed and sedimented onto polylysine-coated coverslips. The samples were subsequently dehydrated in a series of graded ethanol, critical point dried and coated with 2 nm iridium. Cells were analysed by field-emission scanning electron microscopy (SEM) (Carl Zeiss Merlin) using secondary electron detector at accelerating voltage of 5 kV and probe current of 100 pA at 50KX magnification.

Gene expression analysis

We used available RNA-Sequencing data from NCBI's sequence read archive (SRA)[2] to estimate gene expression levels in *V. cholerae*. The data from 307 experiments run on an Illumina sequencing

machine were downloaded and quantified against a decoy-aware index generated from the GCF_000829215.1_ASM82921v1 reference[3] using salmon/v1.3.00[4]. Experiments with less than 50% of reads matching a reference gene were filtered out. The estimated counts were then imported into R/v4.0 using the tximport package[5] and transformed using the varianceStabilizingTransformation procedure implemented in the DESeq2[6] R package to generate a pseudo-log2 count set.

β-galactosidase assay

The putative promoter regions that span the 300 to 500 bp preceding the initial ATG codon of the *vc1313*, *vc1312* and *vc1311* genes were cloned into promoter-probe plasmid pCB192N[7]. Cultures of *V. cholerae* wild-type or Δ*bsrV* mutant carrying pCB192N derivatives with promoter-lacZ transcriptional fusions were started from a 1:1000 dilution of an overnight culture and grown in LB at 37 ºC. Cultures were collected after 8h of growth or at different times, as indicated. β-galactosidase assays were performed[8]. Miller units were calculated from 2 independent experiments containing 3 biological replicates each.

SEC-MALS

Elution peaks of MCP_{DRK}-LBD further analysed by size-exclusion chromatography coupled to multi-angle laser light scattering (SEC-MALS) with the use of an ÄKTApure system (GE Healthcare) coupled to a miniDAWN TREOS II detector and an OptiLab T-rEX online refractive index detector (Wyatt Technology). The absolute molar mass was calculated by analysing the scattering data using the ASTRA analysis software package, version 7.2.2.10 (Wyatt Technology). BSA was used for calibration and proteins were separated on a Superdex 200 Increase 10/300 analytical SEC column (GE Healthcare) with a flow rate of 0.4 ml min^{-1}. For each run a 200 μL fraction of purified MCP_{DRK}-LBD (~0.2 to 2 mg) was injected on the SEC column and eluted in 20 mM HEPES/NaOH (pH 7.8) and 150 mM NaCl. For the runs with D-Arg, 10 mM D-Arg was added to 4 mg ml^{-1} purified MCP_{DRK}-LBD and incubated for 10 minutes at 25°C, before injecting ~100 μL fractions (~0.4 mg) on the SEC column equilibrated in 20 mM HEPES/NaOH (pH 7.8), 150 mM NaCl and 10 μM D-Arg. The refractive index increment of MCP_{DRK}-LBD was set at 0.185 ml g^{-1} and the extinction coefficient for UV detection at 280 nm was calculated from the primary structure of the protein construct.

Supplementary Source Data

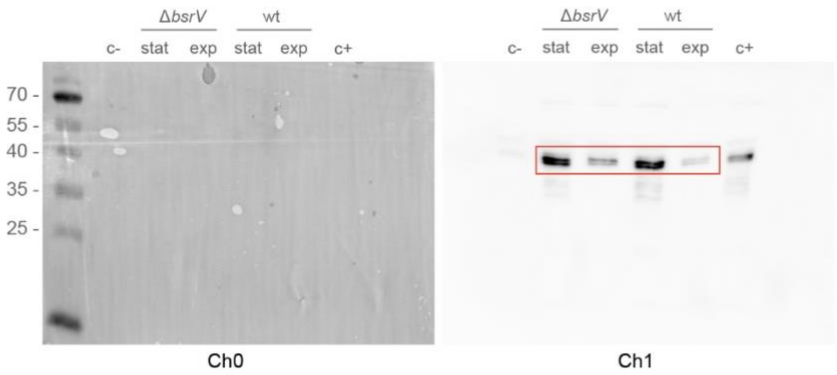

Supplementary Source Data Supplementary Figure 1. Unprocessed blot.

Supplementary references

1. Schindelin, J. *et al.* Fiji: an open-source platform for biological-image analysis. *Nature Methods 2012 9:7* **9**, 676–682 (2012).
2. Leinonen, R., Sugawara, H. & Shumway, M. The sequence read archive. *Nucleic Acids Res* **39**, (2011).
3. Okada, K. et al. Comparative genomic characterization of a Thailand-Myanmar isolate, MS6, of Vibrio cholerae O1 El Tor, which is phylogenetically related to a 'US Gulf Coast' clone. *PLoS One* **9**, (2014).
4. Patro, R., Duggal, G., Love, M. I., Irizarry, R. A. & Kingsford, C. Salmon provides fast and bias-aware quantification of transcript expression. *Nat Methods* **14**, 417–419 (2017).
5. Soneson, C., Love, M. I. & Robinson, M. D. Differential analyses for RNA-seq: transcript-level estimates improve gene-level inferences. *F1000Res* **4**, 1521 (2015).
6. Love, M. I., Huber, W. & Anders, S. Moderated estimation of fold change and dispersion for RNA-seq data with DESeq2. *Genome Biol* **15**, (2014).
7. Kimsey, H. H. & Waldor, M. K. Vibrio cholerae LexA coordinates CTX prophage gene expression. *J Bacteriol* **191**, 6788–6795 (2009).
8. Miller, J. H. Assay of b-galactosidase. *Experiments in molecular genetics* 352–355 (1972).
9. Heidelberg, J. F. *et al.* DNA sequence of both chromosomes of the cholera pathogen Vibrio cholerae. *Nature* **406**, 477–483 (2000).
10. Lam, H. *et al*. D-amino acids govern stationary phase cell wall remodeling in bacteria. *Science (1979)* **325**, 1552–1555 (2009).
11. Donnenberg, M. S. & Kaper, J. B. Construction of an eae deletion mutant of enteropathogenic Escherichia coli by using a positive-selection suicide vector. *Infect Immun* **59**, 4310–4317 (1991).
12. Bina, X. R., Wong, E. A., Bina, T. F. & Bina, J. E. Construction of a tetracycline inducible expression vector and characterization of its use in Vibrio cholerae. *Plasmid* **76**, 87–94 (2014).
13. Cava, F., de Pedro, M. A., Lam, H., Davis, B. M. & Waldor, M. K. Distinct pathways for modification of the bacterial cell wall by non-canonical D-amino acids. *EMBO Journal* **30**, 3442–3453 (2011).
