# Supplementary material for: d-amino acids signal a stress-dependent run-away response in Vibrio cholerae
Source: Nat Microbiol. 2023 Jun 26;8(8):1549–60. doi: 10.1038/s41564-023-01419-6 (PMC10390336; doi:10.1038/s41564-023-01419-6)
Supplement: Supplementary file 2 — Reporting Summary [file 41564_2023_1419_MOESM2_ESM.pdf]

nature portfolio

Corresponding author(s): Felipe Cava

Last updated by author(s): May 23, 2023

Reporting Summary

Nature Portfolio wishes to improve the reproducibility of the work that we publish. This form provides structure for consistency and transparency in reporting. For further information on Nature Portfolio policies, see our Editorial Policies and the Editorial Policy Checklist.

Statistics

For all statistical analyses, confirm that the following items are present in the figure legend, table legend, main text, or Methods section.

n/a	Confirmed	
☐	☒	The exact sample size (*n*) for each experimental group/condition, given as a discrete number and unit of measurement
☐	☒	A statement on whether measurements were taken from distinct samples or whether the same sample was measured repeatedly
☐	☒	The statistical test(s) used AND whether they are one- or two-sided<br>*Only common tests should be described solely by name; describe more complex techniques in the Methods section.*
☒	☐	A description of all covariates tested
☐	☒	A description of any assumptions or corrections, such as tests of normality and adjustment for multiple comparisons
☐	☒	A full description of the statistical parameters including central tendency (e.g. means) or other basic estimates (e.g. regression coefficient) AND variation (e.g. standard deviation) or associated estimates of uncertainty (e.g. confidence intervals)
☐	☒	For null hypothesis testing, the test statistic (e.g. F, t, r) with confidence intervals, effect sizes, degrees of freedom and P value noted<br>*Give P values as exact values whenever suitable.*
☒	☐	For Bayesian analysis, information on the choice of priors and Markov chain Monte Carlo settings
☒	☐	For hierarchical and complex designs, identification of the appropriate level for tests and full reporting of outcomes
☒	☐	Estimates of effect sizes (e.g. Cohen's *d*, Pearson's *r*), indicating how they were calculated

Our web collection on statistics for biologists contains articles on many of the points above.

Software and code

Policy information about availability of computer code

Data collection	Biotek Gen5 [v.08] was used to collect OD600 measurements for growth curves. Fuji LAS-3000 Imaging System was used to collect agar plate images. Zeiss Zen 2 Blue edition [v2.0.0.0] software was used for collection of microscopy data.
Data analysis	Graphpad Prism 8.0 and R package ggplot [v4.0] were used for graphing and analyzing most data. Microscopy images and videos were analyzed with Fiji/ImageJ [v1.53], MicrobeJ plugin [v5.13] and cellProfiles package [v3.0.1]. Phylogenetic analysis was carried out by Jalview [v2.11.2.0] and iTOL [v5]. R [v4.0] and packages salmon [v1.3.00], tximport and DESeq2 were used for analysis of publicly available RNAseq data. Coot [0.9.5] was used to build the models and the structures were refined using Refmac5 [v5.8.0267] and PHENIX refine [v1.13].

For manuscripts utilizing custom algorithms or software that are central to the research but not yet described in published literature, software must be made available to editors and reviewers. We strongly encourage code deposition in a community repository (e.g. GitHub). See the Nature Portfolio guidelines for submitting code & software for further information.

Data

Policy information about availability of data

All manuscripts must include a data availability statement. This statement should provide the following information, where applicable:

- Accession codes, unique identifiers, or web links for publicly available datasets
- A description of any restrictions on data availability
- For clinical datasets or third party data, please ensure that the statement adheres to our policy

The mass spectrometry proteomics data have been deposited to the ProteomeXchange Consortium via the PRIDE partner repository (accession: PXD038312). Atomic coordinates and structure factors of the MCPDRK-LBD crystal structures (in complex with D-Arg (PDB: 8BSA) and D-Lys (PDB:8BSB) have been deposited in the Protein Data Bank.

Human research participants

Policy information about studies involving human research participants and Sex and Gender in Research.

Reporting on sex and gender	N/A
Population characteristics	N/A
Recruitment	N/A
Ethics oversight	N/A

Note that full information on the approval of the study protocol must also be provided in the manuscript.

Field-specific reporting

Please select the one below that is the best fit for your research. If you are not sure, read the appropriate sections before making your selection.

☒ Life sciences ☐ Behavioural & social sciences ☐ Ecological, evolutionary & environmental sciences

For a reference copy of the document with all sections, see nature.com/documents/nr-reporting-summary-flat.pdf

Life sciences study design

All studies must disclose on these points even when the disclosure is negative.

Sample size	Sample size was chosen based on preliminary studies, the available literature and the variability between replicates. No statistical method was used to determine sample size.
Data exclusions	No data was excluded from the study and analyses.
Replication	Experiments described in the manuscript were fully replicated, with three or more biological replicates.
Randomization	Samples were allocated into groups based on the mutant identity of Vibrio cholerae.
Blinding	Blinding was not necessary in our study because it did not include any animals and/or human research participants.

Reporting for specific materials, systems and methods

We require information from authors about some types of materials, experimental systems and methods used in many studies. Here, indicate whether each material, system or method listed is relevant to your study. If you are not sure if a list item applies to your research, read the appropriate section before selecting a response.

Materials & experimental systems

n/a	Involved in the study
☐	☒ Antibodies
☒	☐ Eukaryotic cell lines
☒	☐ Palaeontology and archaeology
☒	☐ Animals and other organisms
☒	☐ Clinical data
☒	☐ Dual use research of concern

Methods

n/a	Involved in the study
☒	☐ ChIP-seq
☒	☐ Flow cytometry
☒	☐ MRI-based neuroimaging

Antibodies

Antibodies used

anti-GFP (Thermo Fisher, ref: A-11122) and anti-FlaA (non-commercial) specific antibodies were used to identify the proteins of interest.

Validation

anti-GFP is commercially available and has been validated by the manufacturer. Any validation statements are available on the manufacturer's website. anti-FlaA was previously validated in literature (PMID: 25989366) and in-house running appropriate controls (flaA knock out and a FlaA+ samples as negative and positive controls, respectively).
