# Supplementary figures and images for: d-amino acids signal a stress-dependent run-away response in Vibrio cholerae
Source: Nat Microbiol. 2023 Jun 26;8(8):1549–60. doi: 10.1038/s41564-023-01419-6 (PMC10390336; doi:10.1038/s41564-023-01419-6)

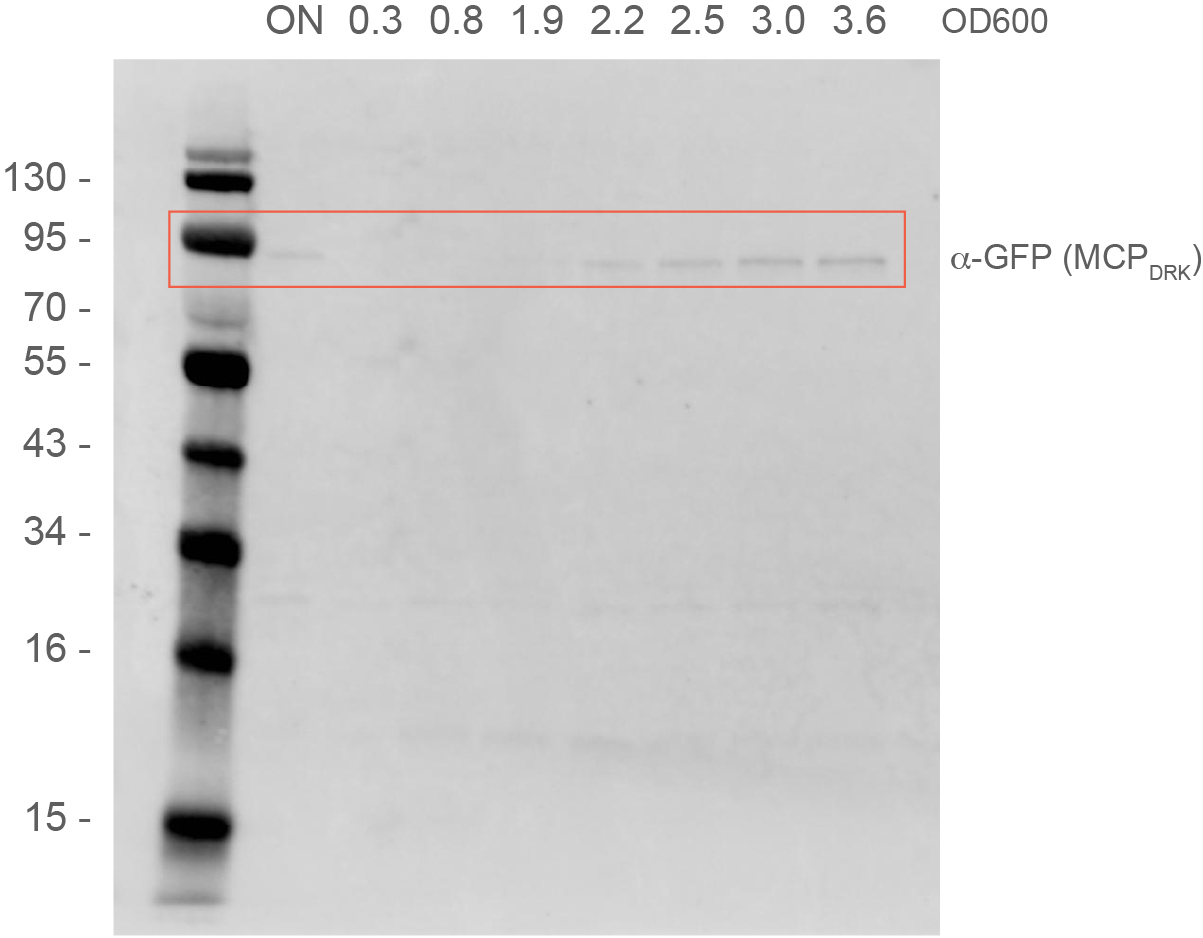

Supplement: Source Data Fig. 1 — Statistical source data. [file 41564_2023_1419_MOESM4_ESM.jpg]
